# Supplementary material for: Identification of scavenger receptor BI as a scavenger of free heme that is essential for protection against hemolysis
Source: Nat Commun. 2026 Jul 27;17:9108. doi: 10.1038/s41467-026-76099-x (PMC13518855; doi:10.1038/s41467-026-76099-x)
Supplement: Supplementary file 1 [file 41467_2026_76099_MOESM1_ESM.pdf]

Major Resources Tables

Mouse Breeding: Albcre mice in C57BL/6J background (Stock No:003574) were obtained from the Jackson laboratory. SR-BI$^{fl/fl}$ mice in 8x C57BL/6J background were from Dr. Chieko Mineo (University of Texas Southwestern Medical Center). AlbcreSR-BI$^{fl/fl}$ and SR-BI$^{fl/fl}$ mice were backcrossed to C57BL/6J for 10 generations.

Supplementary Table 1

	Vendor or Source	Breeding Strategy	Background Strain
**Parent - Male**	In house	SR-BI$^{fl/fl}$ mice	8x C57BL/6J
**Parent - Female**	In house	Albcre mice	C57BL/6J

Supplementary Table 2

Mouse Models (in vivo studies)

Mouse Model	Vendor or Source	Sex
**AlbcreSR-BI$^{fl/fl}$ mice**<br>**SR-BI$^{fl/fl}$ mice**	In house breeding	male & female
**TLR4$^{-/-}$ mice**	Jackson laboratory Stock No:007227	Male & female
**TLR4$^{-/-}$AlbcreSR-BI$^{fl/fl}$**	In house breeding	male & female
**TLR4$^{+/+}$AlbcreSR-BI$^{fl/fl}$**	In house breeding	male & female
**Hba$^{-/-}$Hbb$^{+/-}$Tg$^{+/-}$**	Jackson laboratory Stock No: 003342	male & female
**Hba$^{-/-}$Hbb$^{-/-}$Tg$^{+/+}$**	Jackson laboratory Stock No: 003342	male & female
**Hba$^{-/-}$Hbb$^{-/-}$Tg$^{+/-}$**<br>**Hba$^{-/-}$Hbb$^{-/-}$Tg$^{+/+}$**<br>**Hba$^{-/-}$Hbb$^{+/-}$Tg$^{+/-}$**	In house breeding	male & female

Supplementary Table 3

Mouse Housing Conditions

	Mouse Housing Conditions	Note
**Set Temperature range**	22 °C	
**Set Humidity range**	50%	
**Light Cycle (Mouse)**	14 hours : 10 hours	

**Water**	RO Water	ad libitum
**Standard Feed**	Teklad Irradiated Global 18% Protein Rodent Diet (Envigo); Diet # 2918	ad libitum
**Standard Bedding**	P.J. Murphy Coarse SaniChip	
**Experimental Feed**	Teklad Irradiated Global 18% Protein Rodent Diet (Envigo); Diet # 2918	ad libitum
**SPF**	Yes	

Supplementary Table 4

Major Resources Table

Reagents or Resources	Source	Identifier
**Antibodies**		
**Anti-rabbit IgG (1:2000-5000)**	Jackson Immuno Research	111-035-003
**Anti-mouse IgG (1:5000)**	Cappel	55550
**Anti-sheep IgG (1:1000)**	Sigma	12-342
**Anti-ß-actin (1:10000)**	Sigma	A5441
**Anti-SR-BI (1:2000)**	Sigma-Genosys	customer made
**Anti-HO-1 (1:1000)**	Cell Signaling	E3F4S
**Anti-CD91 (1:50000)**	Abcam	Ab92544
**Anti-Hp (1:500)**	Novus	AF8465
**Anti-Hx (1:2000)**	Novus	AF7007
**LysoTracker**	Thermo Fisher Scientific	Green DND-26
**Critical Commercial Assays**		
**Un-bound Free Hemin Assay Kit**	Sigma	MAK036
**Bicinchoninic acid assay**	Thermo Fisher Scientific	23225
**Chemicals and Recombinant Proteins**		
**APS**	Bio-Rad	161-0700
**Resolving gel buffer 1.5M Tris-HCL PH 8.8**	Bio-Rad	161-0798
**Stacking gel buffer 0.5M Tris-HCL PH 6.8**	Bio-Rad	161-0799
**TEMED**	Bio-Rad	161-0800
**Restore western blot stripping buffer**	Fisher	21059
**30% Acrylamide/Bis solution**	Bio-Rad	161-0156
**Red Taq**	Bioline	bio-25044
**FBS**	Bio-Techne	S11550H; FBS123
**ECL**	Fisher	32106
**Agarose**	Invitrogen	16500-500
**L-glutamine**	Invitrogen	25030-081; S11550H
**Penicillin- Streptomycin**	Invitrogen	151401-22; 546120

**Proteinase K**	Invitrogen	25530-031
**G418**	Sigma	G8168
**Bovine serum albumin (BSA)**	Sigma	A3294; 057K0740
**Hemin**	Sigma	H9039
**Zn(II)Mesoporphyrin IX**	Frontier Scientific	M40628
**Phenylhydrazine hydrochloride (PHZ)**	Sigma	114715
**BLT-1**	Sigma	373210
**Hemopexin, Human Plasma**	Athens Research & Technology	16-16-080513
**Lipoprotein, high density from human plasma**	Sigma	L8039
**Trypan blue**	Sigma	T8154; RNBB8457
**2-Mercaptoethanol**	Sigma	M3148; 103K0081
**Ham's F-12**	CORNING	10-080-CV
**Dulbecco's Modified Eagle Medium**	CORNING	10-017-CV
**Trypsin-EDTA Solution**	Sigma	T4049
**PBS without calcium magnesium**	Sigma	D8537
**Proteinase inhibitor**	Sigma	P2714
**Human SCARB1/CD36L1/SR-BI Protein, With C-Fc Tag**	Acro	SC1-H5258
**Collagen I , rat tail**	Sigma	C7661
**Liberase™ TM Research Grade**	Sigma	5401119001
**Percoll**	Santa Cruz Biotechnology	NC2049819
**Sosium Citrate, 3.8%(W/V)**	Fisher	722816
**ProLong™ Gold Antifade Mountant with DAPI**	Invitrogen™	P36941

Supplementary Table 5

Primers

Gene	Primer
**Albcre**	20239:5′-TGCAAACATCACATGCACAC-3′<br>20240: 5′-TTGGCCCCTTACCATAAC TG-3′<br>oIMR5374: 5′-GAAGCAGAAGCTTAGGAAGATGG-3′
**TLR4**	oIMR8365 GCA AGT TTC TAT ATG CAT TCT C<br>oIMR8366 CCT CCA TTT CCA ATA GGT AG<br>oIMR8367 ATA TGC ATG ATC AAC ACC ACA G<br>oIMR8368 TTT CCA TTG CTG CCC TAT AG
**Hba**	22807 GAC CTA CTT CCC TCA CTT TGA TG<br>22808 CAC TAT GTT CCC TGC CTT GG<br>oIMR1139 ATA GAT GGG TAG CCA TTT AGA TTC C<br>oIMR1140 CCG GGT TAT AAT TAC CTC AGG TC
**Hbb**	12300 AGA ATA GCC AGG GGA AGG AA<br>12316 CTG TCC ATC TGC ACG AGA CT<br>13545 ACC CAC AGC CAA ACA CTA GG

**Tg**	13546 GAA GGG AAG AAG CAA CAT G<br>24905 TCA AGG AGG CAA ATG GAA AC<br>24906 AGG CAC CTA GCA CCT TCT TG<br>oIMR7338 CTA GGC CAC AGA ATT GAA AGA TCT<br>oIMR7339 GTA GGT GGA AAT TCT AGC ATC ATC C
**SR-BI$^{fl/fl}$**	Fwd GCACAGAGGACCCAACAGCGCACAAAATGG<br>Rev: GCTGGGATTCAAGGTGTGTGCCACCACTAC

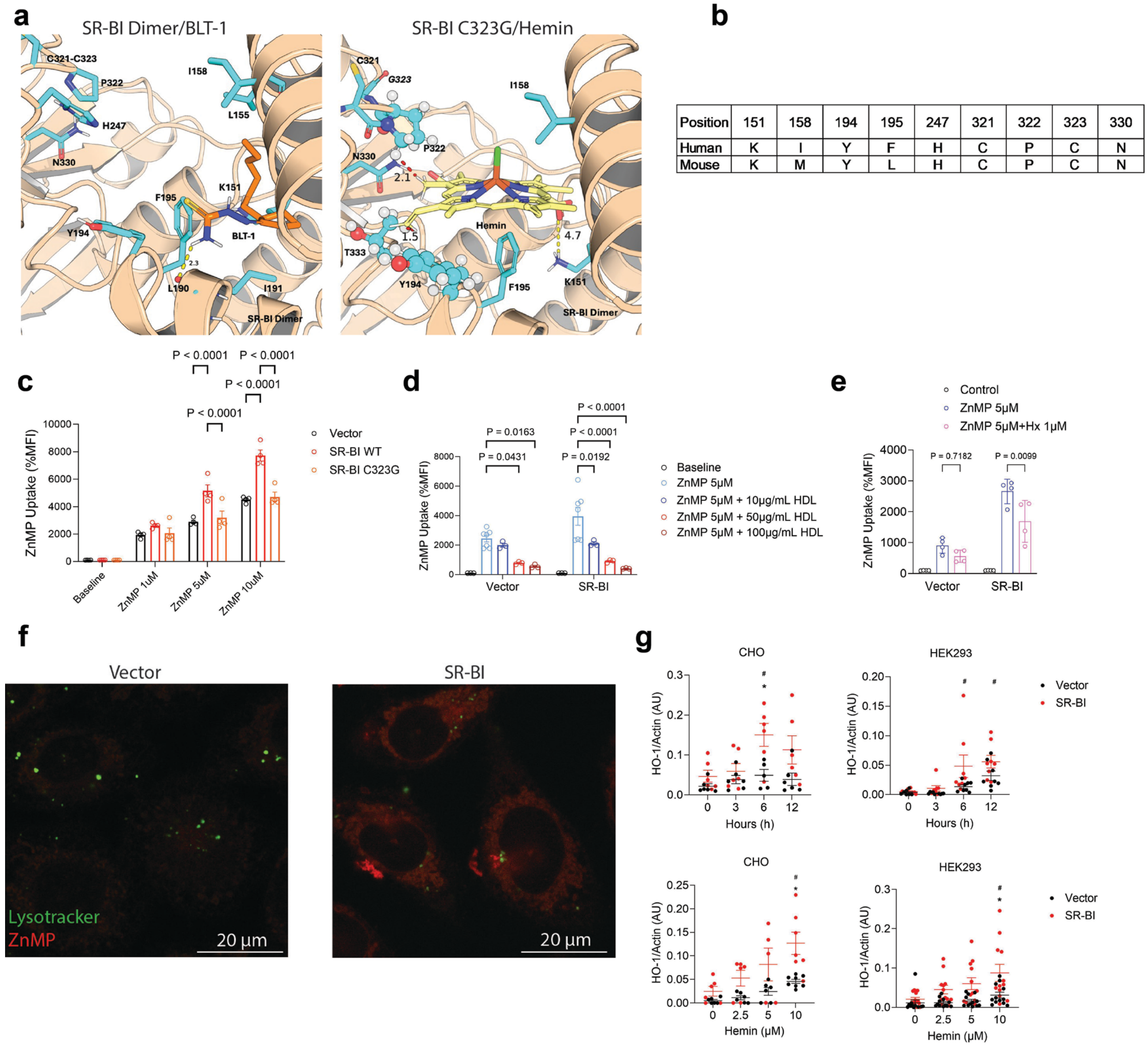

Fig. S1. **SR-BI facilitates the uptake of free heme.**

(**a**) Binding modes of SR-BI WT with BLT-1, and SR-BI C323G mutant with hemin, predicted using an energy-minimization workflow implemented in AutoDock Vina. (**b**) Amino acid sequences similarity between human and mouse SR-BI involved in hemin binding. (**c**) Flow cytometry analysis of ZnMP uptake in CHO cells expressing vector,

SR-BI WT or SR-BI C323G mutant. n=4 independent experiments. (**d**) Flow cytometry analysis of ZnMP uptake in CHO cells expressing vector and SR-BI WT, in the presence of 10, 50, 100 μg/ml high-density lipoprotein (HDL), n=3 independent experiments for HDL inhibition groups, n=7 independent experiments for 5 μM ZnMP control groups; and (**e**) in the presence of 1 μM hemopexin (Hx), n= 4 independent experiments. (**f**) CHO cells expressing vector or SR-BI were incubated with 20 μM ZnMP for 30mins and co-stained with 500 nM LysoTracker for 1hr to assess intracellular localization. (**g**) Individual data point for hemin time and dose-dependent HO-1 expression in Figure 1 (**m-p**). The * denotes comparison with the control at the same time point, while # denotes comparison with baseline. The means ± SEM were plotted. Figure 1 (**m**) 6 hrs Vector vs SR-BI, p=0.0345; SR-BI 6 hrs vs Baseline, p=0.0179. (**n**) SR-BI 6 hrs vs Baseline, p=0.0154; SR-BI 12 hrs vs Baseline, p=0.0021. (**o**) SR-BI 10 μM hemin vs Baseline, p=0.0007; 10 μM hemin Vector vs SR-BI, p=0.0053. (**p**) SR-BI 10 μM hemin vs Baseline, p=0.0012; 10 μM hemin Vector vs SR-BI, p=0.011.; Significances were determined by two-way ANOVA.

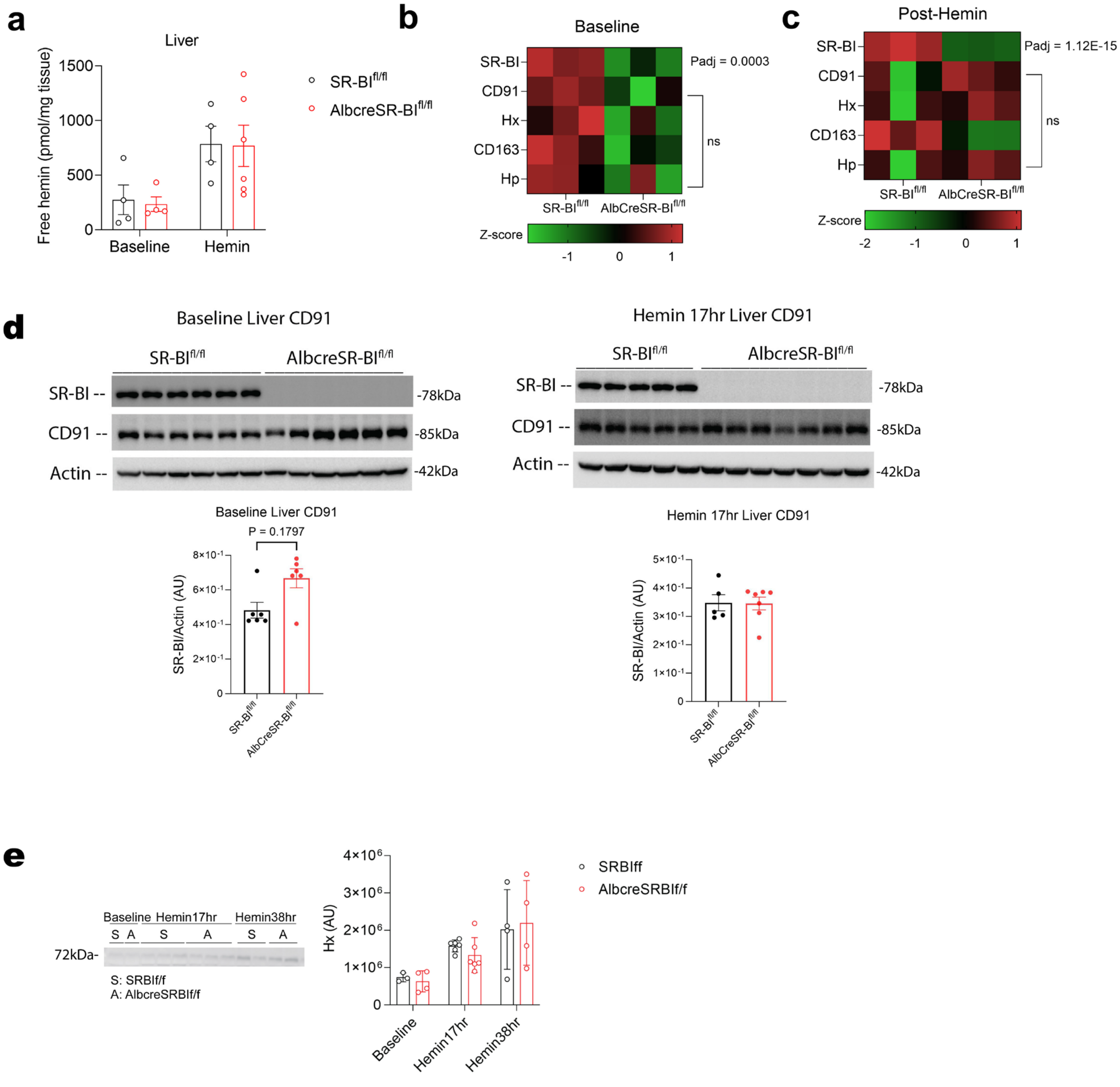

Fig. S2. Liver gene profiles of SR-BI$^{fl/fl}$ and AlbcreSR-BI$^{fl/fl}$ mice.

(**a**) Free hemin levels in the liver tissue lysates were measured one hour post the 2nd hemin injection from the free hemin clearance study. SR-BI$^{fl/fl}$ (n=4) and AlbcreSR-BI$^{fl/fl}$ (n=4 under baseline condition; n=6 under hemin condition). (**b-c**) Heatmap of genes of interests from SR-BI$^{fl/fl}$ and AlbcreSR-BI$^{fl/fl}$ mice liver (n=3) at baseline and after hemin treatment. None significant (ns) is based on adjusted p-value. DESeq2 framework and Benjamini-Hochberg FDR were used for multiple testing correction. (**d**) SR-BI$^{fl/fl}$ and AlbcreSR-BI$^{fl/fl}$ mice at baseline and after one injection of 40 µmol/kg BW of hemin (i.p.). SR-BI$^{fl/fl}$ (n=6 under baseline condition; n=5 under hemin condition) and AlbcreSR-BI$^{fl/fl}$ (n=6 under baseline condition; n=7 under hemin condition). (**e**) SR-BI$^{fl/fl}$ and AlbcreSR-BI$^{fl/fl}$ mice at baseline and after one injection of 40 µmol/kg BW of hemin (i.p.). SR-BI$^{fl/fl}$ (n=3 under baseline condition; n=6 under hemin 17hrs condition; n=4 under hemin

38hrs condition) and AlbcreSR-BI$^{fl/fl}$ (n=4 under baseline condition; n=6 under hemin 17hrs condition; n=4 under hemin 38hrs condition). 30 µg of liver lysate and plasma protein were loaded and examined for SR-BI, CD91, Actin, and Hx via immunoblot. The means ± SEM were plotted. Significance was determined by two-way ANOVA for (**a,e**) and Mann-Whitney test (**d**). Data represents n = X biological replicates (individual mice), with no technical replicates used for statistical testing.

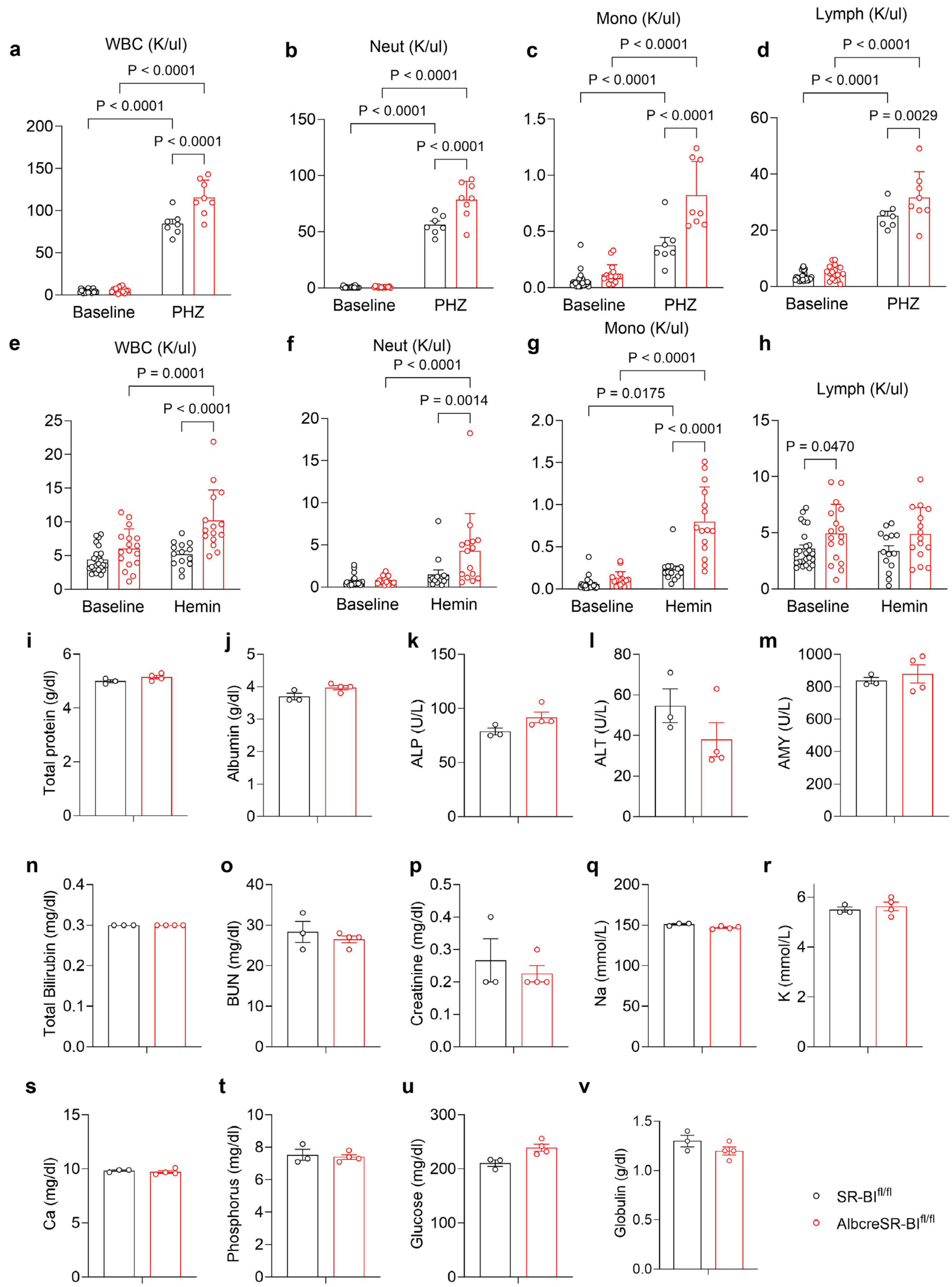

Fig. S3. CBC in mice treated with PHZ and hemin. Baseline biochemistry panel

Total white blood cells (WBC), neutrophil (Neut), monocytes (Mono), lymphocytes (Lymph) in SR-BI$^{fl/fl}$ and AlbcreSR-BI$^{fl/fl}$ mice after intraperitoneal injections with PHZ 30 mg/kg BW (4 shots) (**a-d**) and hemin 40 μmol/kg BW (5 shots) (**e-h**). SR-BI$^{fl/fl}$ (n=24 under baseline condition; n=7 under PHZ condition; n=14 under hemin condition) and AlbcreSR-BI$^{fl/fl}$ (n=17 under baseline condition; n=8 under PHZ condition; n=15 under hemin condition). (**i-v**) Biochemistry panel of SR-BI$^{fl/fl}$ (n=3) and AlbcreSR-BI$^{fl/fl}$ (n=4) at baseline. Means ± SEM were plotted. Significances were determined by two-way ANOVA for (**a-h**) and Mann-Whitney for (**i-v**). Data represents n = X biological replicates (individual mice), with no technical replicates used for statistical testing.

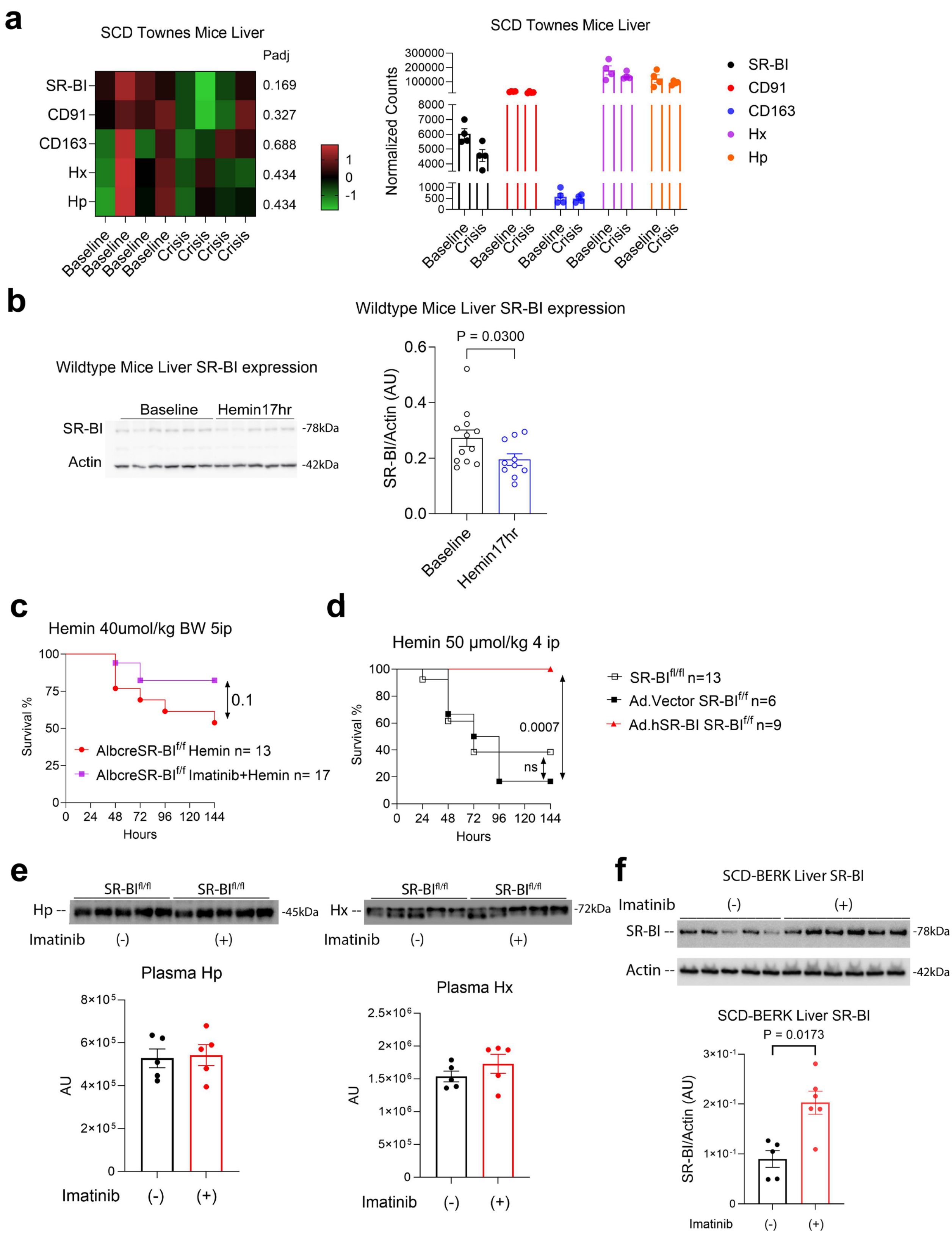
a
SCD Townes Mice Liver
Padj
SR-BI 0.169
CD91 0.327
CD163 0.688
Hx 0.434
Hp 0.434
1
0
-1
Baseline Baseline Baseline Baseline Crisis Crisis Crisis Crisis
SCD Townes Mice Liver
Normalized Counts
SR-BI
CD91
CD163
Hx
Hp
b
Wildtype Mice Liver SR-BI expression
Baseline Hemin17hr
SR-BI -78kDa
Actin -42kDa
Wildtype Mice Liver SR-BI expression
P = 0.0300
SR-BI/Actin (AU)
Baseline
Hemin17hr
c
Hemin 40umol/kg BW 5ip
Survival %
0.1
AlbcreSR-BI f/f Hemin n= 13
AlbcreSR-BI f/f Imatinib+Hemin n= 17
Hours
d
Hemin 50 μmol/kg 4 ip
Survival %
0.0007
ns
Hours
SR-BI fl/fl n=13
Ad.Vector SR-BI f/f n=6
Ad.hSR-BI SR-BI f/f n=9
e
SR-BI fl/fl SR-BI fl/fl
Hp -45kDa
Imatinib (-) (+)
SR-BI fl/fl SR-BI fl/fl
Hx -72kDa
Imatinib (-) (+)
Plasma Hp
AU
Imatinib (-) (+)
Plasma Hx
AU
Imatinib (-) (+)
f
SCD-BERK Liver SR-BI
Imatinib (-) (+)
SR-BI -78kDa
Actin -42kDa
SCD-BERK Liver SR-BI
P = 0.0173
SR-BI/Actin (AU)
Imatinib (-) (+)

Fig. S4.

(**a**) Heatmap and normalized count of hemolysis scavenger gene expression at the liver of SCD Townes mice n=4 (Public Dataset). (**b**) SR-BI$^{fl/fl}$ mice were injected with 40 μmol/kg BW of hemin (i.p.). 30 μg of liver lysate were loaded and examined for SR-BI level via immunoblot. (n =12 for baseline condition; n=10 for Hemin 17hrs condition). (**c**) Survival analysis of AlbcreSR-BI$^{fl/fl}$ mice treated with/without imatinib (50 mg/kg BW) for 3 days, followed by daily hemin injection. (**d**) Survival analysis of SR-BI$^{fl/fl}$ mice treat with no adenovirus, Ad.hSR-BI or Ad.vector for 3 days, followed by daily hemin injection. (**e**) Plasma Hp and Hx level in SR-BI$^{fl/fl}$ mice (n=5) treated with/without imatinib for 3 days. 30 μg of plasma protein were loaded and examined for Hp and Hx via immunoblot. (**f**) SCD-BERK mice were treated with (n=6) or without imatinib (n=5) (50 mg/kg BW) for 3 days. 30 μg of liver lysate were loaded and examined for SR-BI level via immunoblot. Survival curves were analyzed by the Log-Rank test. The means ± SEM were plotted. Significance was determined by Mann-Whitney. Data represents n = X biological replicates (individual mice), with no technical replicates used for statistical testing.

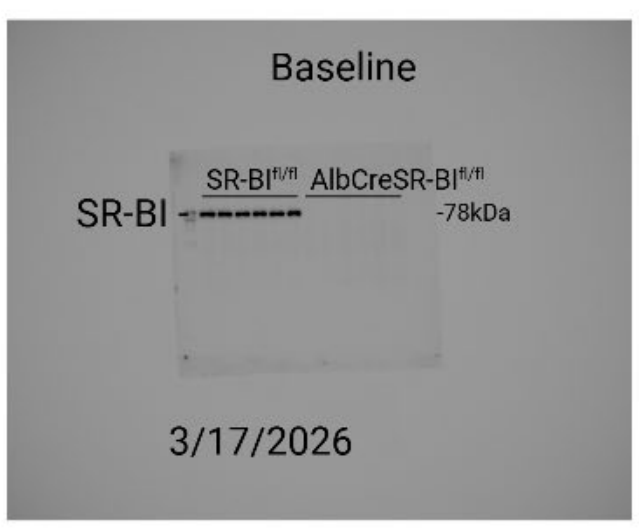

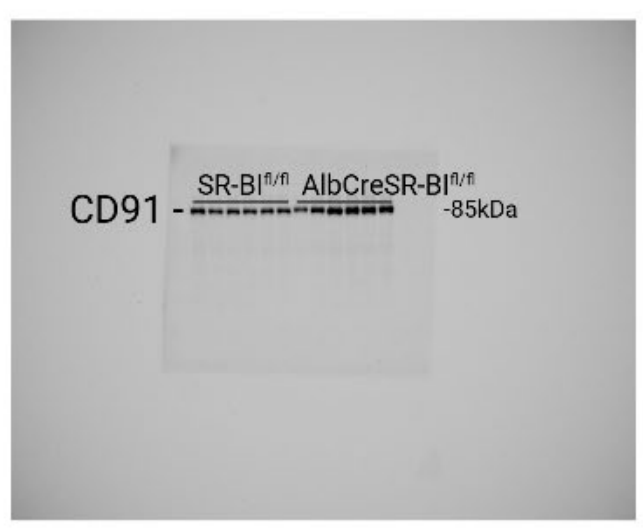

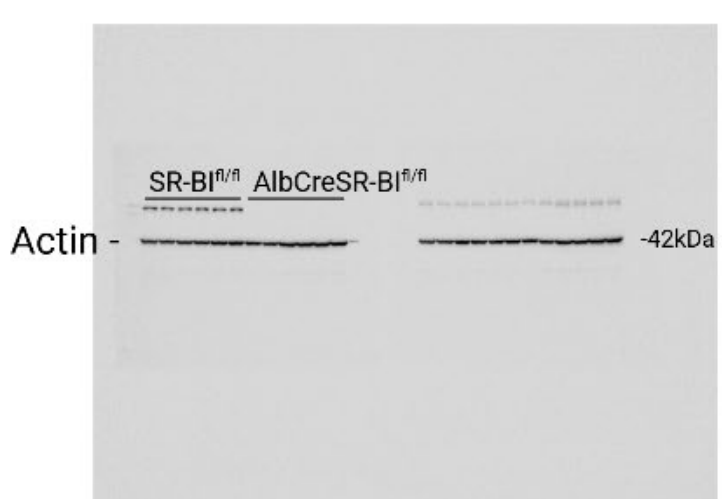

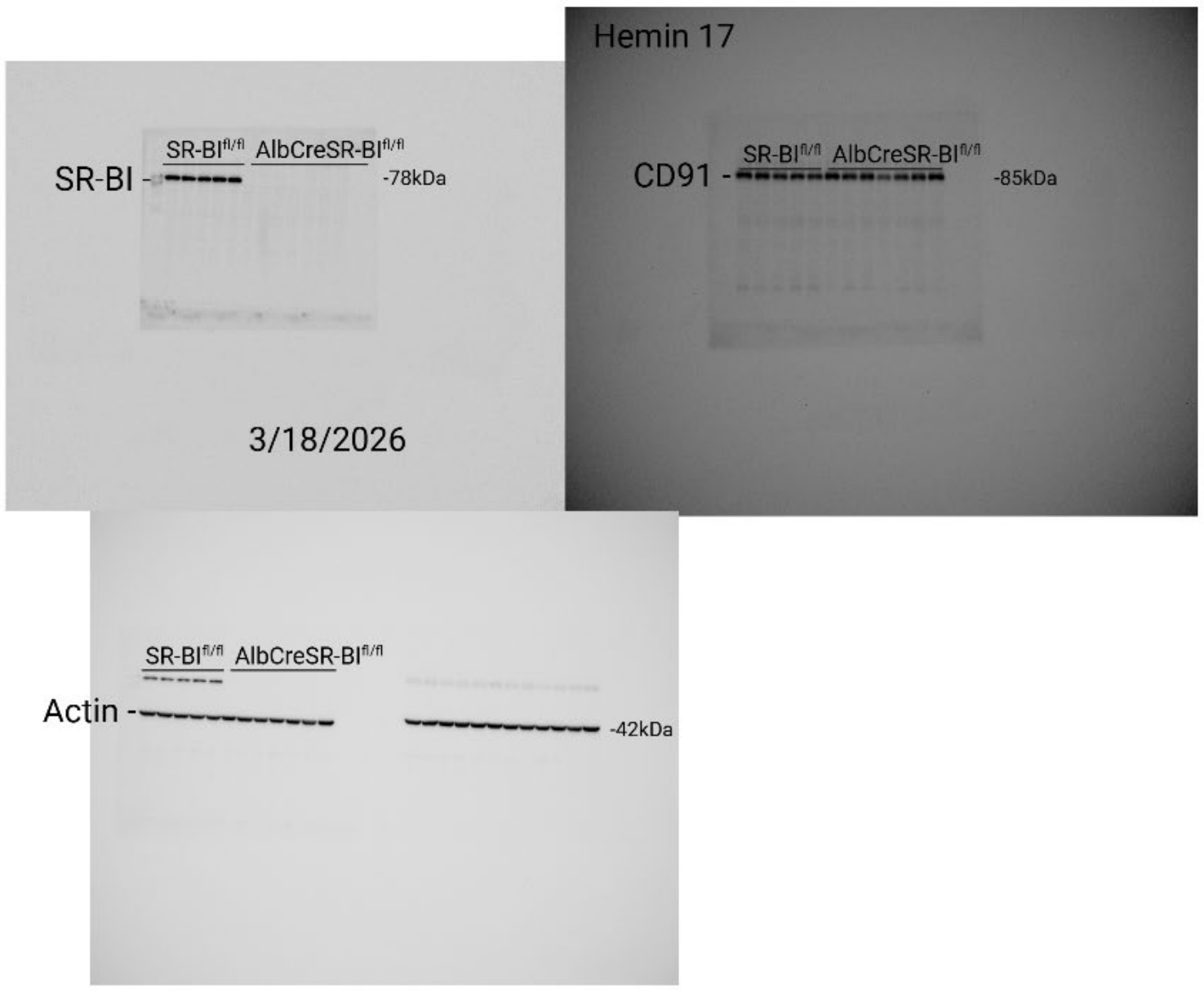

Extended Fig2d

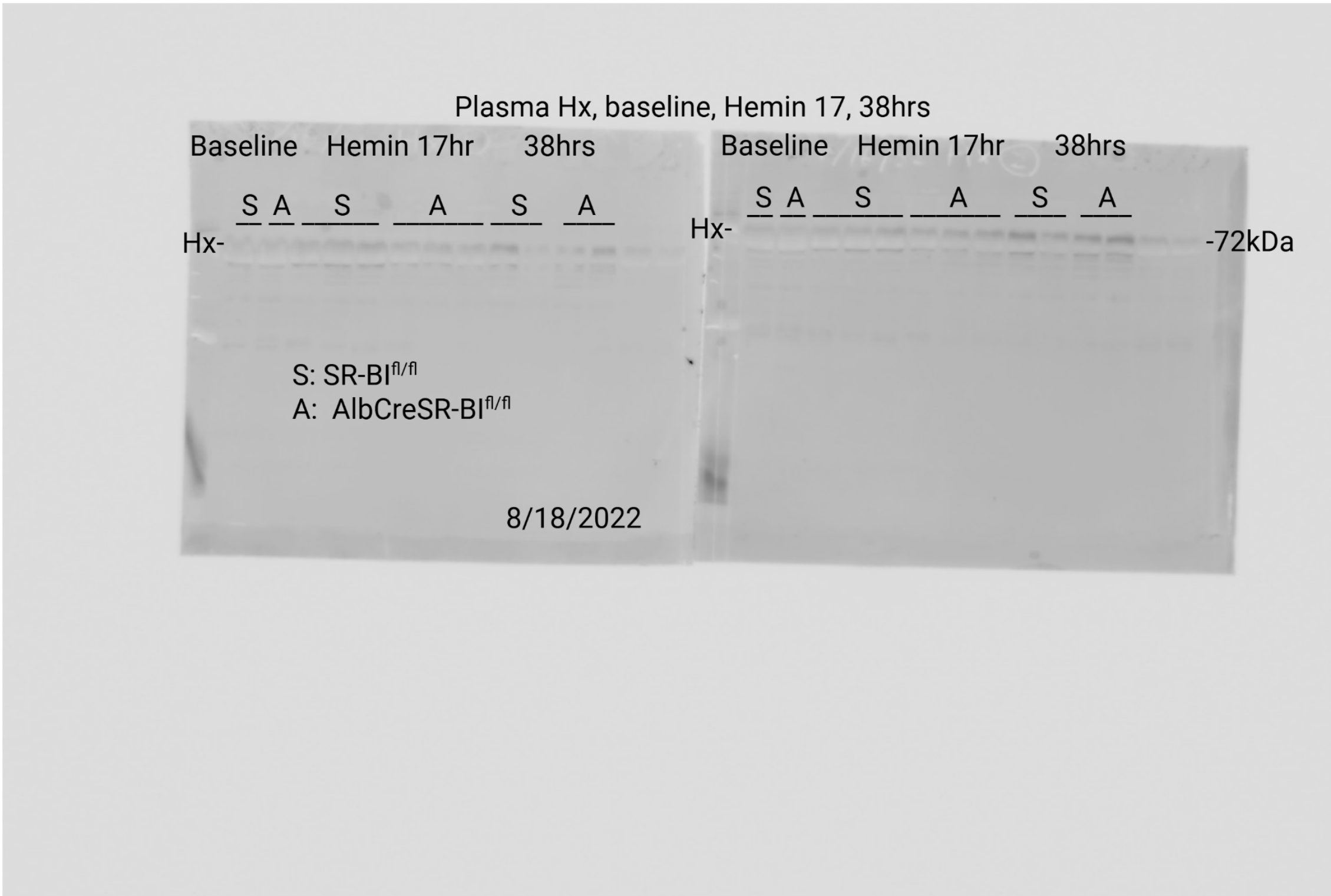

Extended Fig2e

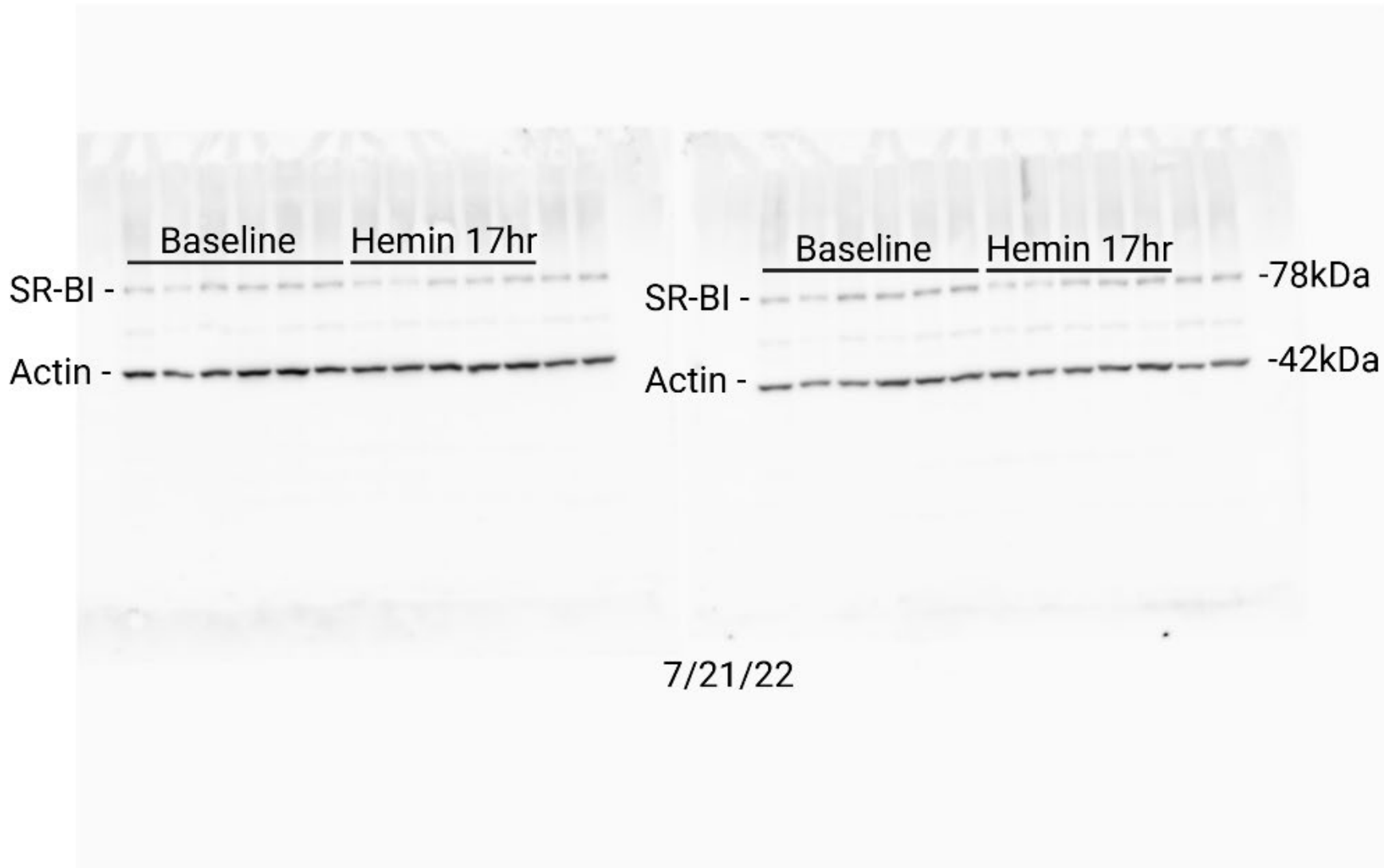

Extended Fig4b

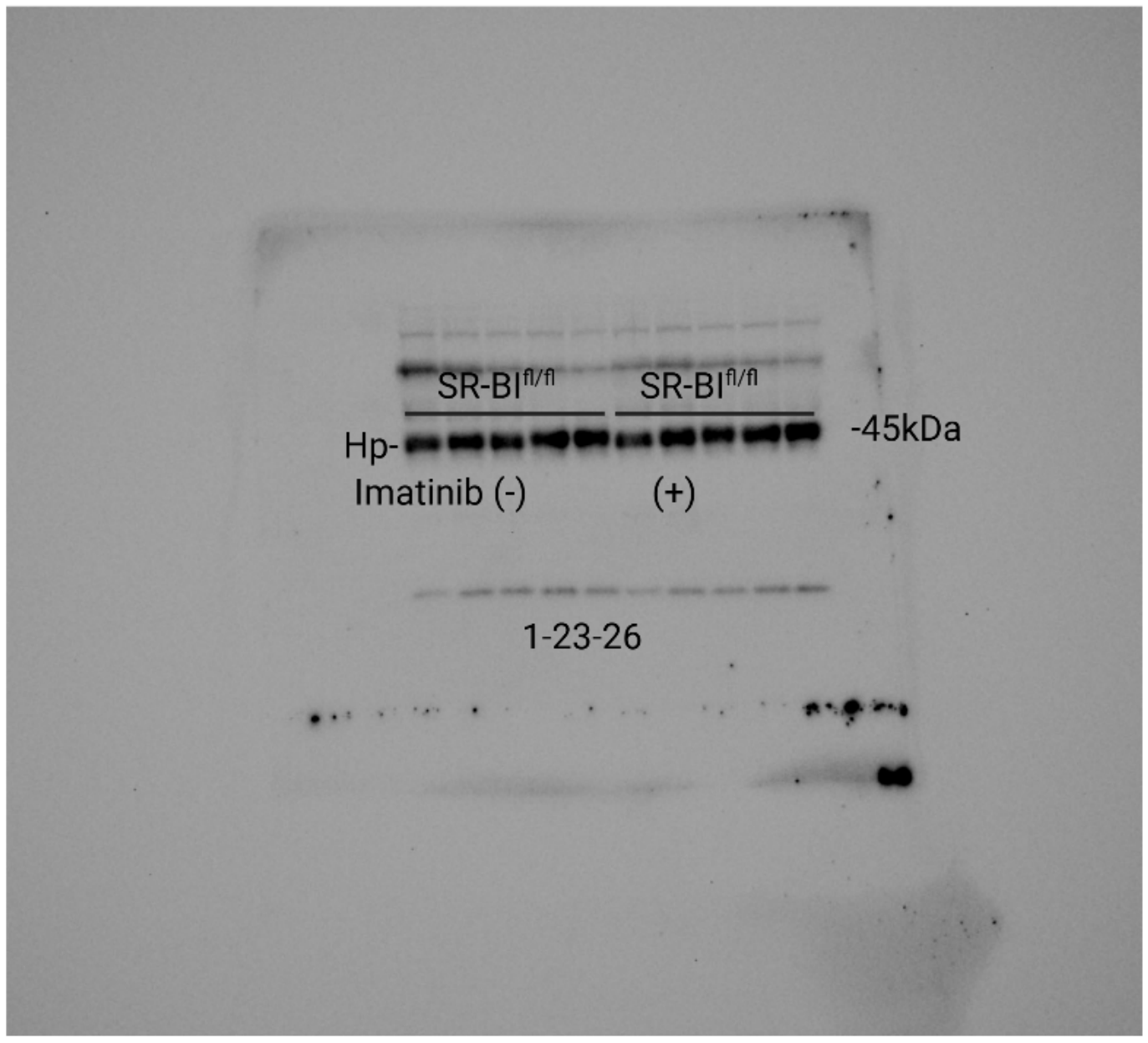

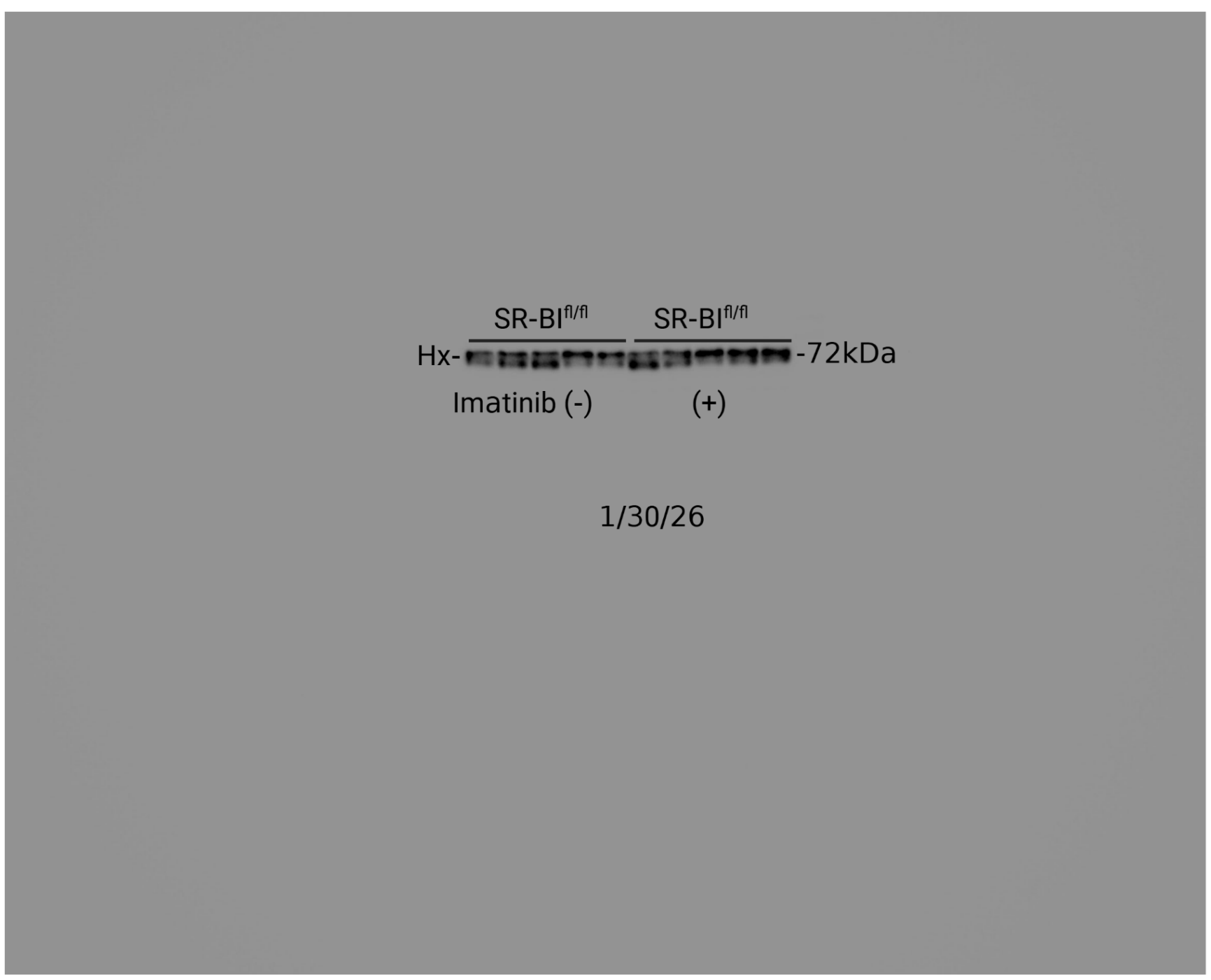

Extended Fig4e

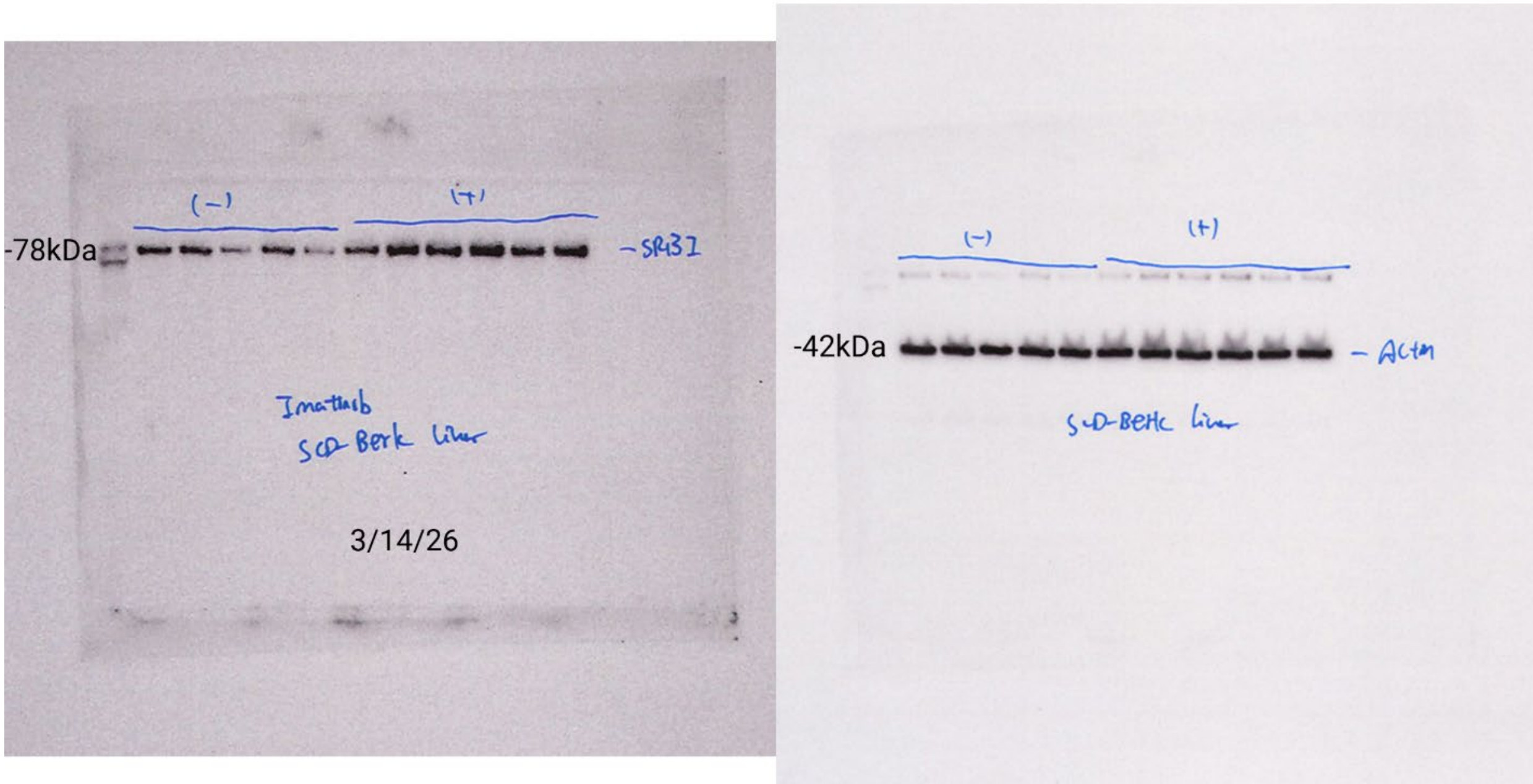

Extended Fig4f
