# Supplementary material for: Identification of scavenger receptor BI as a scavenger of free heme that is essential for protection against hemolysis
Source: Nat Commun. 2026 Jul 27;17:9108. doi: 10.1038/s41467-026-76099-x (PMC13518855; doi:10.1038/s41467-026-76099-x)
Supplement: Supplementary file 3 [file 41467_2026_76099_MOESM3_ESM.pdf]

nature portfolio

Peer Review File

Identification of scavenger receptor BI as a scavenger of free heme that is essential for protection against hemolysis

Corresponding Author: Professor Xiang-an Li

Parts of this Peer Review File have been redacted as indicated to remove third-party material.

This file contains all reviewer reports in order by version, followed by all author rebuttals in order by version.

A version of this paper was originally rejected for publication by Nature Communications, however that decision was reconsidered after appeal by the authors.

Version 0:

Reviewer comments:

Reviewer #1

(Remarks to the Author)
The authors present evidence that SR-BI mediates pathological heme uptake and represents a potential therapeutic target in hemolytic conditions. The in vivo protection data are particularly strong. However, several key experiments are needed to establish the mechanism of SR-BI-mediated heme clearance and its relationship to canonical scavenger pathways: (1) validation using authentic Fe-heme to confirm that SR-BI transports physiological heme, not just fluorescent surrogates like ZnMP; (2) subcellular fractionation to determine whether internalized heme reaches the cytosol for HO-1 mediated degradation or is sequestered in lysosomes; and (3) genetic loss-of-function validation demonstrating that therapeutic interventions require SR-BI. Additionally, more rigorous quantitative analysis of canonical scavenger pathway expression would clarify whether these systems compensate for SR-BI loss. Addressing these points would significantly strengthen the mechanistic foundation and therapeutic implications of this work.
Major Concerns
1. The reliance on ZnMP as the sole fluorescent surrogate throughout the uptake experiments is limiting. While ZnMP is convenient for live-cell imaging, it lacks the iron center and associated coordination chemistry that defines heme's biological properties. Questions: does SR-BI transport physiological Fe-protoporphyrin IX with similar kinetics and affinity as observed with ZnMP? To establish the physiological relevance of these findings, the authors should validate their key observations using direct Fe-heme. This could be accomplished preferably through ^{55}Fe-heme uptake assays, which are well-established in the heme biology field. Alternatively, through heme-specific biochemical quantification methods such as the pyridine hemochrome assay following cell lysis. At minimum, the authors should demonstrate in a representative experiment (e.g., CHO-SR-BI cells) that authentic Fe-heme uptake parallels the ZnMP uptake patterns, and that SR-BI overexpression similarly enhances Fe-heme internalization. Without this validation, it remains uncertain whether the therapeutic effects observed in vivo genuinely result from SR-BI-mediated Fe-heme clearance or whether ZnMP uptake represents a related but distinct phenomenon.
2. Where does the internalized heme accumulate within cells? This is important as it is proposed that SR-BI-mediated heme uptake leads to HO-1 induction and cytoprotective heme degradation. However, HO-1 is a cytosolic/ER-associated enzyme, meaning productive heme degradation requires cytosolic delivery. If SR-BI-mediated heme uptake instead results in lysosomal sequestration, this would represent a fundamentally different mechanism (cellular compartmentalization rather than enzymatic degradation). Authors should perform subcellular fractionation experiments to determine the distribution of internalized heme across cytosolic, membrane, and lysosomal fractions. Co-localization studies using standard organelle markers (e.g., LAMP1 for lysosomes, cytosolic markers) would complement this analysis. Demonstrating that heme reaches the cytosol would strongly support the proposed mechanism, whereas lysosomal accumulation would require re-interpretation of how SR-BI provides protection. This distinction is essential for understanding the therapeutic mechanism and predicting efficacy in different disease contexts.
3. The authors show that imatinib treatment and adenoviral SR-BI overexpression protect mice from heme-induced lethality. However, the SR-BI-dependence of this protection has not been genetically validated. Imatinib has multiple cellular targets beyond SR-BI regulation, and adenoviral delivery itself can trigger cellular responses. To confirm that the observed protection is specifically mediated by SR-BI, the authors should test whether these interventions lose efficacy in AlbcreSR-

BIfl/fl mice. Specifically, do imatinib-treated or adenovirus-treated SR-BI knockout mice show any protection compared to vehicle-treated knockout controls? This experiment is essential to establish SR-BI as the mechanism of action and to support the therapeutic rationale. If protection is completely lost in knockouts, this strongly validates SR-BI as the target. If partial protection remains, this will indicate additional SR-BI–independent mechanisms that should be identified and discussed.

4. The authors conclude that Hp-Hb-CD163 and Hx-Hp-CD91 systems operate primarily under physiological conditions, while SR-BI is selectively induced for pathological heme loads. However, Figure S2 presents heatmap data with n=3 per group, which provides limited statistical power to detect changes in these pathways. The heatmap format makes it difficult to assess whether observed differences are statistically significant. To test whether canonical scavenger pathways are unchanged or potentially compensatory, the authors should provide quantitative expression data (RT-qPCR or Western blot) with appropriate statistical analysis for Hp, Hx, CD163, and CD91 in wild-type versus SR-BI knockout livers at baseline and following hemin challenge. If these pathways show significant compensatory upregulation in SR-BI knockouts, this will indicate functional redundancy that could explain why SR-BI knockout mice retain substantial heme clearance capacity, which would be important for interpreting the relative contribution of SR-BI and the therapeutic potential of SR-BI modulation.

Minor Suggestions

5. HO-1 is used as an indicator of heme uptake and SR-BI function. While HO-1 is indeed heme-responsive, it is also induced by oxidative stress, inflammatory signals, and Nrf2 activation independent of heme. The authors should clarify whether HO-1 induction specifically correlates with measurable heme uptake. If the subcellular fractionation experiments suggested above are performed, correlating cytosolic heme levels with HO-1 expression would strengthen the interpretation that HO-1 induction reflects functional heme delivery rather than a general stress response to hemolysis.

6. The acute hemin injection model effectively demonstrates SR-BI's capacity to protect against sudden, high heme loads. However, sickle cell disease and other chronic hemolytic conditions involve sustained, moderate hemolysis over extended periods. To strengthen the translational relevance, the authors could test whether SR-BI upregulation (via imatinib or other means) provides protection in a chronic SCD mouse model (e.g., Townes SCD mice) by assessing markers of organ damage, inflammation, and survival over weeks to months. This would establish whether the therapeutic benefit extends beyond acute heme toxicity to chronic hemolytic disease states, which is critical for clinical translation.

7. To better understand how SR-BI integrates with existing heme detoxification systems, the authors could measure whether Hp and Hx levels are altered following therapeutic SR-BI upregulation. If SR-BI operates as an independent cellular uptake pathway that works downstream of or in parallel to plasma scavengers, SR-BI upregulation might not affect Hp/Hx consumption. Conversely, if these systems are functionally coupled, SR-BI upregulation might accelerate clearance of Hp-heme or Hx-heme complexes. Additionally, characterizing Hp, Hx, and SR-BI expression levels in SCD models during steady state versus acute crisis would reveal whether SR-BI is selectively induced when canonical scavengers become saturated or depleted, providing insight into when SR-BI-targeted therapy might be most beneficial. Lastly, knockout of Hp and Hx to solidify SR-BI in pathological heme clearance will be useful.

Reviewer #2

(Remarks to the Author)

Comments to the authors

Ito and colleagues identify the scavenger receptor BI (SR-BI) as a mediator of free heme clearance. The findings are novel and of clinical relevance, with important implications for disorders associated with hemolysis. This reviewer has a few suggestions for additional experiment for the author's consideration. Additionally, the data analyses should be reviewed and likely revised as to ensure appropriate statistical analysis of the data

1. The authors point out that the SR-BI binds to heme with low affinity with a Kd at 18.2 µM determined with SPR. The authors should provide addition information from the kinetic traces. Did the authors determine association and dissociation rates?

2. Apart from computational modeling, the authors should consider confirming the heme/SR-BI interaction using other methods – NMR, cryo-EM

3. Additionally, kinetic experiments using receptor mutants of predicted binding sites could strengthen the results

4. Did the authors conduct SPR with reversed orientation? Different immobilization levels? Those experiment could improve the interpretation of the results and shed light on how such low affinity interaction yield the physiologic responses reported.

5. The experiments shown in Figure S1 examining the effect of HDL on ZnMP uptake should include a dose response both of HDL and ZnMP. A dose-response experiment would greatly add to the understanding of how do already known SR-BI ligands affect heme binding.

6. Figure 2 panels b and c – note that blood is collected from the mice 4 times over a period of 60 min. How much is drawn each time? What are the implications of phlebotomy on plasma hemin concentration and free hemin clearance? Was the volume replaced? In those figures what analyses were performed – the figure legend indicates that unpaired Mann-Whitney test for hemin clearance. This is not appropriate as there are two factors that might affect hemin levels: the model (SR-BIfl/fl and Albcre SR-BIfl/fl) and time – a two-way repeated measures ANOVA is more appropriate rather than unpaired Mann-Whitney test for hemin clearance as the legend intimates.

7. Did the authors measure free hemin in the liver in the experiments described in Figure 2d and 2e?

8. What hepatic and metabolic alterations do Albcre SR-BIfl/fl mice present? If any could those interfere on the animals' susceptibility to hemin?

9. In the model of sickle cell disease, non-SCD littermates may not be the ideal controls as these animals express mouse hemoglobin. The authors should use the HbAA – BERK mice that akin to the SCD-BERK do not express any mouse hemoglobin genes but human hemoglobin A.

10. Imatinib has been shown to have beneficial effects in SCD mice. The authors should consider an experiment evaluating

the effect of imatinib on SR-BI expression and mortality in hemin -challenged SCD mice. This would add to the translational potential of the study
11. The experiments involving administration of Ad.Vector and AdhSRBI should include control mice (SR-BIfl/fl, in figure 4e and non-SCD and HbAA-BERK controls in figure 4H). How do we know there is no untoward effect of Ad.Vector injections
12. Please revise the statistical analysis. Longitudinal data with repeated measures should be accounted for in the analysis. Please show the data points for each mouse in repeated measures experiments – Figure 1n-p; Figure 2 B and 2C

Minor point,

1. Please provide spleen to body weight ratio rather than spleen weight alone as to control for differences in mouse sizes

Reviewer #3

(Remarks to the Author)
Identification of scavenger receptor BI as a scavenger of free heme that is essential for
protection against hemolysis
Summary of the key results:

Ito et al. describe the role scavenger receptor BI (SR-BI) in free heme clearance by the liver. This is demonstrated by addition of free heme in a mouse model lacking SR-BI, resulting in significant heme toxicity, and by upregulating SR-BI with imatinib. They next demonstrate that hepatic expression of SR-BI is reduced in a sickle mouse model with hepatopathy, and that adenoviral mediated upregulation of SR-BI reduced heme toxicity in the sickle mouse model,

Originality and significance: SR-BI may be a drug target to reduce heme toxicity in SCD and other hemolytic conditions. This has been known for over a decade. Others have shown that imatinib reduced lung, kidney, and liver injury in a sickle mouse model, and its impact on an enzyme involved in heme catabolism. This paper does provide some new information regarding the SR-BI mechanism

Data & Methodology: Currently, there is little novel information. The sickle mouse is treated with a heavy hand; there should not be a need to add lethal amounts of hemin to see an effect of adenovirus upregulation of SR-BI. The finding that SR-BI is downregulated in SCD, a disease model that needs to clear heme, is interesting but not explored further.

Suggested improvements: This paper would be improved by more translational work; the upregulation of SR-BI by adenovirus in the sickle mouse model is a good proof of principle, but a drug study, particularly one with a record of use in humans, would bring this close to human use. RNASeq would be more informative in a disease model rather than a mouse model lacking SR-BI

Clarity and context: This paper could benefit from more discussion of diseases in which heme is a driver of clinical complications, and a more complete discussion of therapeutics already in this space.

Version 1:

Reviewer comments:

Reviewer #1

(Remarks to the Author)
I thank the authors for providing additional data and clarifications which have improved the quality and clarity of their manuscript. I do not have additional comments or questions.

Reviewer #2

(Remarks to the Author)
The authors responded to the reviewers' critiques satisfactorily

Reviewer #3

(Remarks to the Author)
The authors have been very responsive to reviewer comments for the most part, while not entirely addressing concerns about fitness as a SCD therapeutic. Since they have acknowledged this in their discussion, this is not a barrier to

publication.

Open Access This Peer Review File is licensed under a Creative Commons Attribution 4.0 International License, which permits use, sharing, adaptation, distribution and reproduction in any medium or format, as long as you give appropriate credit to the original author(s) and the source, provide a link to the Creative Commons license, and indicate if changes were made.
In cases where reviewers are anonymous, credit should be given to 'Anonymous Referee' and the source.
The images or other third party material in this Peer Review File are included in the article's Creative Commons license, unless indicated otherwise in a credit line to the material. If material is not included in the article's Creative Commons license and your intended use is not permitted by statutory regulation or exceeds the permitted use, you will need to obtain permission directly from the copyright holder.
To view a copy of this license, visit https://creativecommons.org/licenses/by/4.0/

Dear Reviewers,

Thank you for your time and effort in reviewing our manuscript. We greatly value the constructive critiques and appreciate the favorable assessment regarding its novelty, high clinical relevance, and strong in vivo data. In response to your insightful suggestions, we performed numerous additional experiments, addressed all concerns in detail, and substantially revised the manuscript. These efforts have significantly strengthened the rigor, clarity, and overall impact of the study.

Below, please find our point-by-point response:

Reviewer 1 (Remarks to the Author):

The authors present evidence that SR-BI mediates pathological heme uptake and represents a potential therapeutic target in hemolytic conditions. The in vivo protection data are particularly strong. However, several key experiments are needed to establish the mechanism of SR-BI-mediated heme clearance and its relationship to canonical scavenger pathways: (1) validation using authentic Fe-heme to confirm that SR-BI transports physiological heme, not just fluorescent surrogates like ZnMP; (2) subcellular fractionation to determine whether internalized heme reaches the cytosol for HO-1 mediated degradation or is sequestered in lysosomes; and (3) genetic loss-of-function validation demonstrating that therapeutic interventions require SR-BI. Additionally, more rigorous quantitative analysis of canonical scavenger pathway expression would clarify whether these systems compensate for SR-BI loss. Addressing these points would significantly strengthen the mechanistic foundation and therapeutic implications of this work.

Major Concerns:

1. The reliance on ZnMP as the sole fluorescent surrogate throughout the uptake experiments is limiting. While ZnMP is convenient for live-cell imaging, it lacks the iron center and associated coordination chemistry that defines heme's biological properties. Questions: does SR-BI transport physiological Fe-protoporphyrin IX with similar kinetics and affinity as observed with ZnMP? To establish the physiological relevance of these findings, the authors should validate their key observations using direct Fe-heme. This could be accomplished preferably through ^{55}Fe-heme uptake assays, which are well-established in the heme biology field. Alternatively, through heme-specific biochemical quantification methods such as the pyridine hemochrome assay following cell lysis. At minimum, the authors should demonstrate in a representative experiment (e.g., CHO-SR-BI cells) that authentic Fe-heme uptake parallels the ZnMP uptake patterns, and that SR-BI overexpression similarly enhances Fe-heme internalization. Without this validation, it remains uncertain whether the therapeutic effects observed in vivo genuinely result from SR-BI-mediated Fe-heme clearance or whether ZnMP uptake represents a related but distinct phenomenon.

--- We thank the reviewer for raising this important concern regarding the use of ZnMP as a fluorescent surrogate for Fe-heme and for the opportunity to clarify our rationale.

We agree that ^{55}Fe-hemin assays have been widely used to study heme uptake. At the outset of this project, we planned to use ^{55}Fe-hemin; however, due to environmental and safety restrictions, our university has been phasing out radioactive materials and related infrastructure. As a result, the scintillation-based detection system required for these assays (Beckman LS6500) was surplused by our department several years ago and is no longer available. We therefore sought an alternative, non-radioactive approach.

We selected ZnMP for several reasons. First, heme transporters and receptors primarily recognize the porphyrin macrocycle rather than the central metal ion. Structural and biophysical studies have demonstrated that substitution of iron with zinc preserves the porphyrin scaffold and overall molecular geometry[1]. Consequently, ZnMP and hemin exhibit identical binding properties to their cognate receptors. In side-by-side comparisons, multiple studies have demonstrated that ZnMP and ^{55}Fe-hemin display quantitatively indistinguishable uptake kinetics and comparable transporter dependence[2-5]. Second, we performed structural modeling to directly compare interactions between SR-BI and either Fe-hemin or ZnMP. As shown in the **Response Letter** (**RL**) **Figure 1**, ZnMP engages SR-BI in a manner highly similar to Fe-hemin, preserving key coordinating and hydrophobic interactions with the porphyrin macrocycle. The predicted binding affinities are nearly identical, with closely matched calculated Gibbs free energies (−111 kcal/mol for hemin versus −110 kcal/mol for ZnMP). These results support the structural and energetic equivalence of ZnMP and Fe-hemin in the context of SR-BI binding. Together, these experimental and computational data support the conclusion that ZnMP serves as a valid structural and functional analog for Fe-hemin in our uptake studies. We have revised the manuscript to (i) cite prior studies demonstrating comparable uptake behavior of ZnMP and Fe-hemin, and (ii) include new side-by-side docking simulations of SR-BI with hemin and ZnMP (revised **Manuscript Figure 1c**).

Importantly, our conclusions do not rely solely on ZnMP-based flow cytometry or fluorescence imaging. We employed multiple independent and complementary approaches, including: (1) direct measurement of SR-BI binding affinity to Fe-hemin using surface plasmon resonance (SPR); (2) validation of hemin uptake in two independent cell systems (CHO and HEK cells), assessed by induction of heme oxygenase-1 (HO-1), a well-established physiological marker of intracellular heme uptake[6]; and (3) in vivo studies in hepatic SR-BI–deficient mice demonstrating impaired heme clearance, increased susceptibility to hemolysis, and heme-induced canonical and inflammatory gene expression patterns consistent with published reports[6-8].

We also carefully evaluated pyridine hemochromogen as a potential non-radioactive alternative. Based on the sensitivity parameters reported by Marcero et al[9], the limit of quantitation of the assay is ~1 μM heme in a 1 mL sample. While this level is appropriate for hemoglobinized cells (e.g., MEL, K562 leukemia cell lines induced to

express globin), it is not compatible with CHO cells, whose heme content is magnitudes lower[10,11]. Therefore, the sensitivity of this assay may not be suitable for reliable quantification of heme uptake in our cell system.

In summary, convergent evidence from structural modeling, biochemical binding assays, cellular uptake analyses, and in vivo phenotypes collectively supports the conclusion that SR-BI directly binds and mediates hemin uptake and clearance. We have incorporated the new docking analysis into the Results section (**Figure 1**) and revised the text accordingly (pages 2–3, lines 85–96 & 102-103).

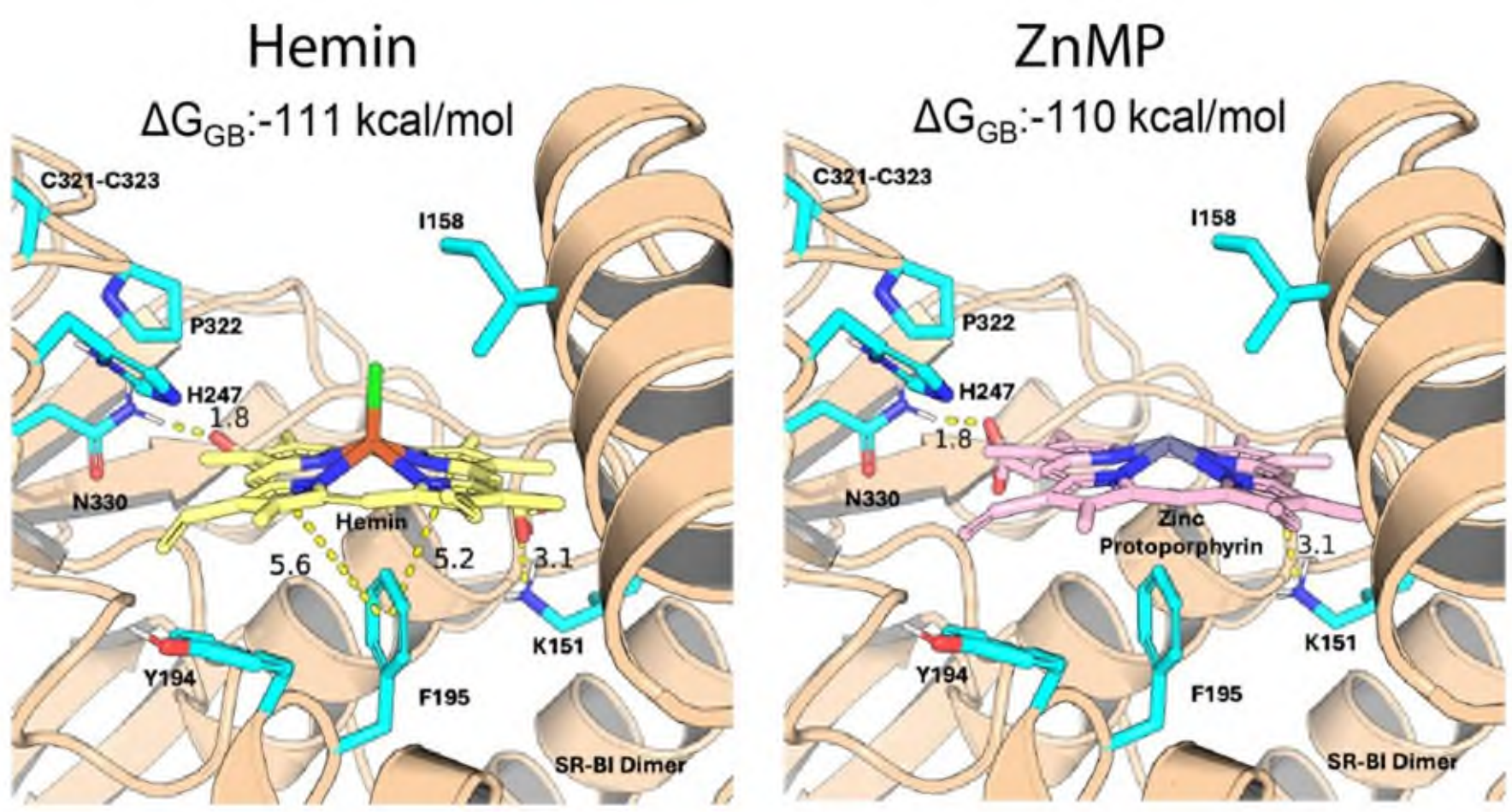

Figure 1. Binding modes of hemin and ZnMP with SR-BI predicted using an energy-minimization workflow implemented in AutoDock Vina.

2. Where does the internalized heme accumulate within cells? This is important as it is proposed that SR-BI-mediated heme uptake leads to HO-1 induction and cytoprotective heme degradation. However, HO-1 is a cytosolic/ER-associated enzyme, meaning productive heme degradation requires cytosolic delivery. If SR-BI-mediated heme uptake instead results in lysosomal sequestration, this would represent a fundamentally different mechanism (cellular compartmentalization rather than enzymatic degradation). Authors should perform subcellular fractionation experiments to determine the distribution of internalized heme across cytosolic, membrane, and lysosomal fractions. Co-localization studies using standard organelle markers (e.g., LAMP1 for lysosomes, cytosolic markers) would complement this analysis. Demonstrating that heme reaches the cytosol would strongly support the proposed mechanism, whereas lysosomal accumulation would require re-interpretation of how SR-BI provides protection. This distinction is essential for understanding the therapeutic mechanism and predicting efficacy in different disease contexts.

--- We thank the reviewer for this important mechanistic point. To address whether SR-BI–mediated heme uptake results in cytosolic/ER-proximal delivery versus lysosomal sequestration, we performed colocalization studies using LysoTracker as a lysosomal marker. As shown in **RL Figure 2** (**Supplemental Figure S1**), internalized

ZnMP exhibits a broad, diffuse distribution throughout the cytosol surrounding the nucleus, with minimal overlap with LysoTracker-positive compartments. This pattern indicates that ZnMP taken up via SR-BI does not predominantly accumulate in lysosomes but instead reaches non-lysosomal compartments consistent with cytosolic and ER-proximal localization. These findings support a model in which SR-BI–mediated heme uptake delivers heme to intracellular sites accessible to HO-1. Importantly, this interpretation is reinforced by our functional data demonstrating robust HO-1 induction in CHO–SR-BI cells exposed to hemin in both time- and dose-dependent manners, consistent with productive heme degradation by the cytosolic/ER-associated HO-1 enzyme. We have incorporated these new data and interpretations into the <u>Results section Page 4, lines 136–145 and included the colocalization analysis as Supplemental Figure S1.</u>

We thank the reviewer for this valuable suggestion. These new data further strengthen our conclusion that SR-BI mediates cellular heme uptake.

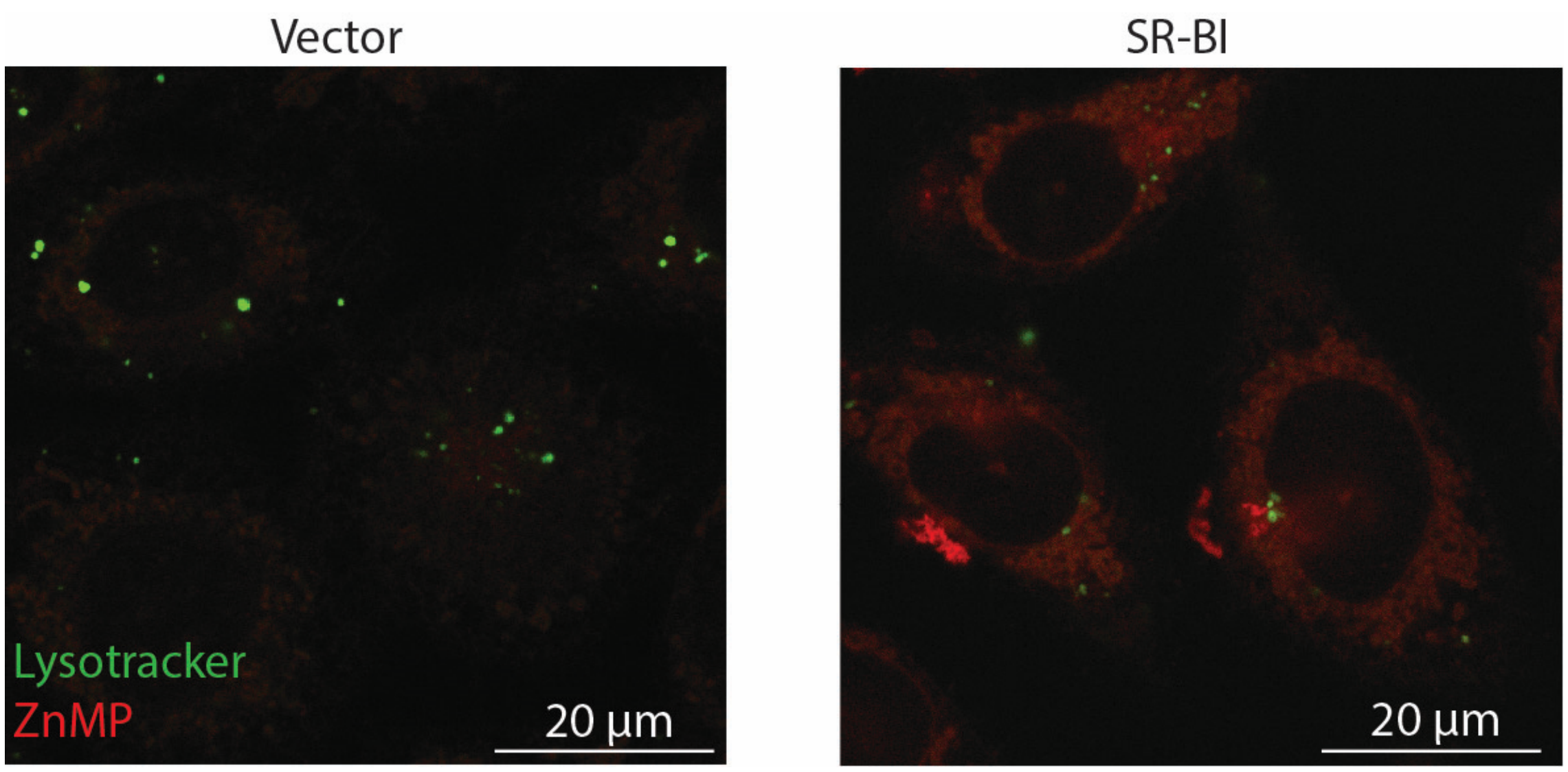

Figure 2. CHO cells expressing vector or SR-BI were incubated with 20 µM ZnMP for 30mins and co-stained with 500nM LysoTracker for 1hr to assess intracellular localization.

3. The authors show that imatinib treatment and adenoviral SR-BI overexpression protect mice from heme-induced lethality. However, the SR-BI-dependence of this protection has not been genetically validated. Imatinib has multiple cellular targets beyond SR-BI regulation, and adenoviral delivery itself can trigger cellular responses. To confirm that the observed protection is specifically mediated by SR-BI, the authors should test whether these interventions lose efficacy in AlbcreSR-BIfl/fl mice. Specifically, do imatinib-treated or adenovirus-treated SR-BI knockout mice show any protection compared to vehicle-treated knockout controls? This experiment is essential to establish SR-BI as the mechanism of action and to support the therapeutic rationale. If protection is completely lost in knockouts, this strongly validates SR-BI as the target. If

partial protection remains, this will indicate additional SR-BI–independent mechanisms that should be identified and discussed.

--- Following your suggestion, we performed additional experiments using AlbCreSR-BI$^{fl/fl}$ mice (**RL Figure 3**). When AlbCreSR-BIfl/fl mice were challenged with lethal doses of hemin, imatinib no longer conferred a survival benefit, indicating that SR-BI is a key mediator of its protective effect. We have added this new data to the Results Section, Pages 9-10 Line 321 to 332 and **supplemental figure S4c**.

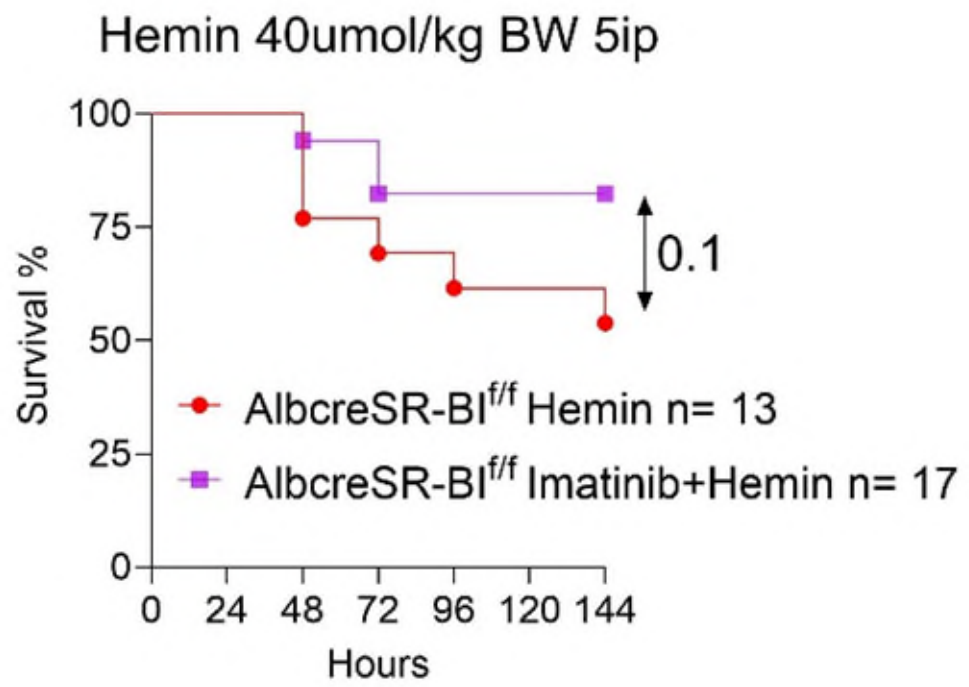

Figure 3. Survival analysis of AlbcreSR-BI$^{fl/fl}$ mice treated with/without imatinib (50 mg/kg BW) for 3 days, followed by daily hemin injection. Survival curves were analyzed by the Log-Rank test.

4. The authors conclude that Hp-Hb-CD163 and Hx-Hp-CD91 systems operate primarily under physiological conditions, while SR-BI is selectively induced for pathological heme loads. However, Figure S2 presents heatmap data with n=3 per group, which provides limited statistical power to detect changes in these pathways. The heatmap format makes it difficult to assess whether observed differences are statistically significant. To test whether canonical scavenger pathways are unchanged or potentially compensatory, the authors should provide quantitative expression data (RT-qPCR or Western blot) with appropriate statistical analysis for Hp, Hx, CD163, and CD91 in wild-type versus SR-BI knockout livers at baseline and following hemin challenge. If these pathways show significant compensatory upregulation in SR-BI knockouts, this will indicate functional redundancy that could explain why SR-BI knockout mice retain substantial heme clearance capacity, which would be important for interpreting the relative contribution of SR-BI and the therapeutic potential of SR-BI modulation.

--- Following your suggestion, we quantified CD91 and hemopexin (Hx) protein levels in wild-type and SR-BI knockout mice at baseline and following hemin challenge. As shown in the below figure, both CD91 and Hx levels did not differ significantly between genotypes at baseline or at post-hemin time point (**RL Figure 4**). Given that Hx is an acute phase protein, we also examined Hx level 38 hours after hemin injection (late crisis, resolution phase) (**RL Figure 5**). We also did not detect significant difference between wild-type and SR-BI knockout mice. These data indicate that the canonical

heme scavenger genes (CD91 and Hx) are not upregulated in SR-BI–deficient livers and therefore does not compensate for loss of SR-BI during acute hemin exposure.

Together, these results demonstrate that SR-BI knockout mice do not activate compensatory Hx upregulation. We thank the reviewer for this thoughtful comment and have added these results to the Result page 6, line 227 to 232 and supplemental figure S2.

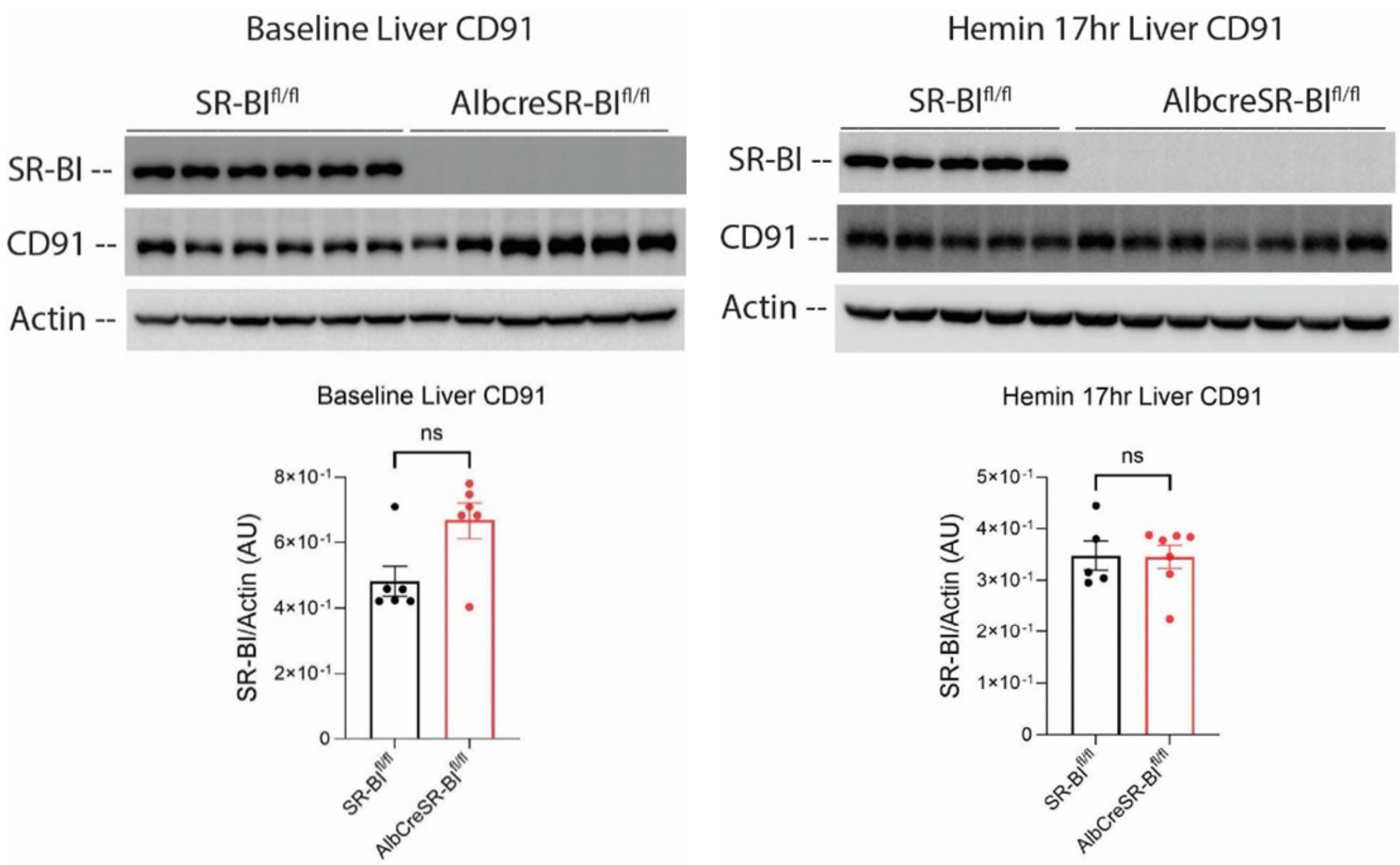

Figure 4. Livers were collected at baseline and 17 hrs post-hemin injection at 40 µmol/kg BW of hemin (i.p.) from SR-BI$^{fl/fl}$ and AlbcreSR-BI$^{fl/fl}$ mice (n = 5-7). 30 µg of liver lysate were loaded and examined for SR-BI, CD91 and β-Actin via immunoblot. Significance was determined by Mann-Whitney test.

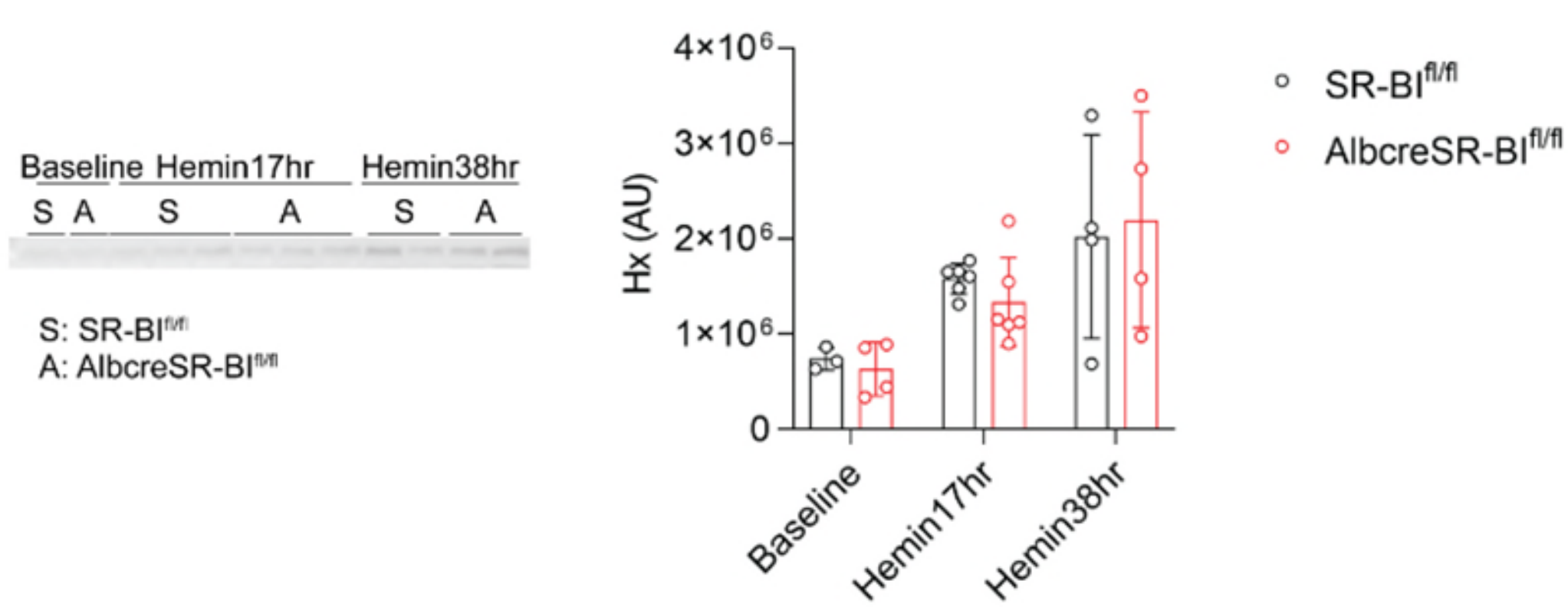

Figure 5. Plasma were collected at baseline, 17, and 38 hrs post-hemin injection at 40 µmol/kg BW of hemin (i.p.) from SR-BI$^{fl/fl}$ and AlbcreSR-BI$^{fl/fl}$ mice (n = 3-6). 30 µg of plasma protein were loaded and examined for Hx via immunoblot. Significance was determined by two-way ANOVA.

Minor Suggestions:

5. HO-1 is used as an indicator of heme uptake and SR-BI function. While HO-1 is indeed heme-responsive, it is also induced by oxidative stress, inflammatory signals, and Nrf2 activation independent of heme. The authors should clarify whether HO-1 induction specifically correlates with measurable heme uptake. If the subcellular fractionation experiments suggested above are performed, correlating cytosolic heme levels with HO-1 expression would strengthen the interpretation that HO-1 induction reflects functional heme delivery rather than a general stress response to hemolysis.

--- We agree that HO-1 is a pleiotropically inducible stress-response gene. Indeed, serum starvation and membrane damage due to oxidative stress and lipid peroxidation of the cell membranes by heme can both lead to HO-1 expression[12,13]. Consistent with these prior studies, our vector control cells also showed enhanced HO-1 expression at 6 and 12 hours of hemin incubation with serum-free media and at 5-10 µM hemin. While HO-1 expression was induced via these unspecific mechanisms in the vector control cells, cells with SR-BI expression showed significantly higher HO-1 expression at the same timepoint and hemin concentration. Consistent with our confocal imaging results where ZnMP was diffused distributed across ER-proximal site that is relevant for HO-1 induction, the robust upregulation of HO-1 supports a specific mechanism rather than a nonspecific stress response. The specific HO-1 induction due to heme uptake via SR-BI is further supported that at baseline, cells expressing SR-BI show no elevation in HO-1 expression compared to vector controls, indicating that SR-BI expression alone does not activate HO-1. We thank the reviewer for raising this important clarification and have added them to the Results section page 4 and line 136 to 145.

6. The acute hemin injection model effectively demonstrates SR-BI's capacity to protect against sudden, high heme loads. However, sickle cell disease and other chronic hemolytic conditions involve sustained, moderate hemolysis over extended periods. To strengthen the translational relevance, the authors could test whether SR-BI upregulation (via imatinib or other means) provides protection in a chronic SCD mouse model (e.g., Townes SCD mice) by assessing markers of organ damage, inflammation, and survival over weeks to months. This would establish whether the therapeutic benefit extends beyond acute heme toxicity to chronic hemolytic disease states, which is critical for clinical translation.

--- We fully agree that testing SR-BI upregulation in a chronic SCD model (e.g., Townes SCD mice) to assess organ damage, inflammation, and even longevity would be highly informative. Indeed, we aim to investigate this in our future studies, which we view as an important but a distinct project from the current manuscript. Our goal here is to establish

SR-BI as a mechanistic determinant of heme handling and acute heme tolerance, which we believe is a necessary foundation for future work.

In the revised manuscript, we added acknowledgment of this limitation in the Discussion section page 13, lane 435 to 445 and highlighted chronic SCD models as a critical next step to test whether SR-BI–targeted strategies (including pharmacologic upregulation) can provide sustained protection in chronic hemolytic disease states.

7. To better understand how SR-BI integrates with existing heme detoxification systems, the authors could measure whether Hp and Hx levels are altered following therapeutic SR-BI upregulation. If SR-BI operates as an independent cellular uptake pathway that works downstream of or in parallel to plasma scavengers, SR-BI upregulation might not affect Hp/Hx consumption. Conversely, if these systems are functionally coupled, SR-BI upregulation might accelerate clearance of Hp-heme or Hx-heme complexes. Additionally, characterizing Hp, Hx, and SR-BI expression levels in SCD models during steady state versus acute crisis would reveal whether SR-BI is selectively induced when canonical scavengers become saturated or depleted, providing insight into when SR-BI-targeted therapy might be most beneficial. Lastly, knockout of Hp and Hx to solidify SR-BI in pathological heme clearance will be useful.

--- Following your suggestion, we measured plasma Hp and Hx after therapeutic SR-BI upregulation. Immunoblots show that Hp and Hx levels remain largely unchanged (**RL Figure 6**), suggesting that hepatic SR-BI operates as an independent heme uptake mechanism. Thank you for raising this point. We added this new data to Results section page 10, line 328 to 332 and supplemental figure S4.

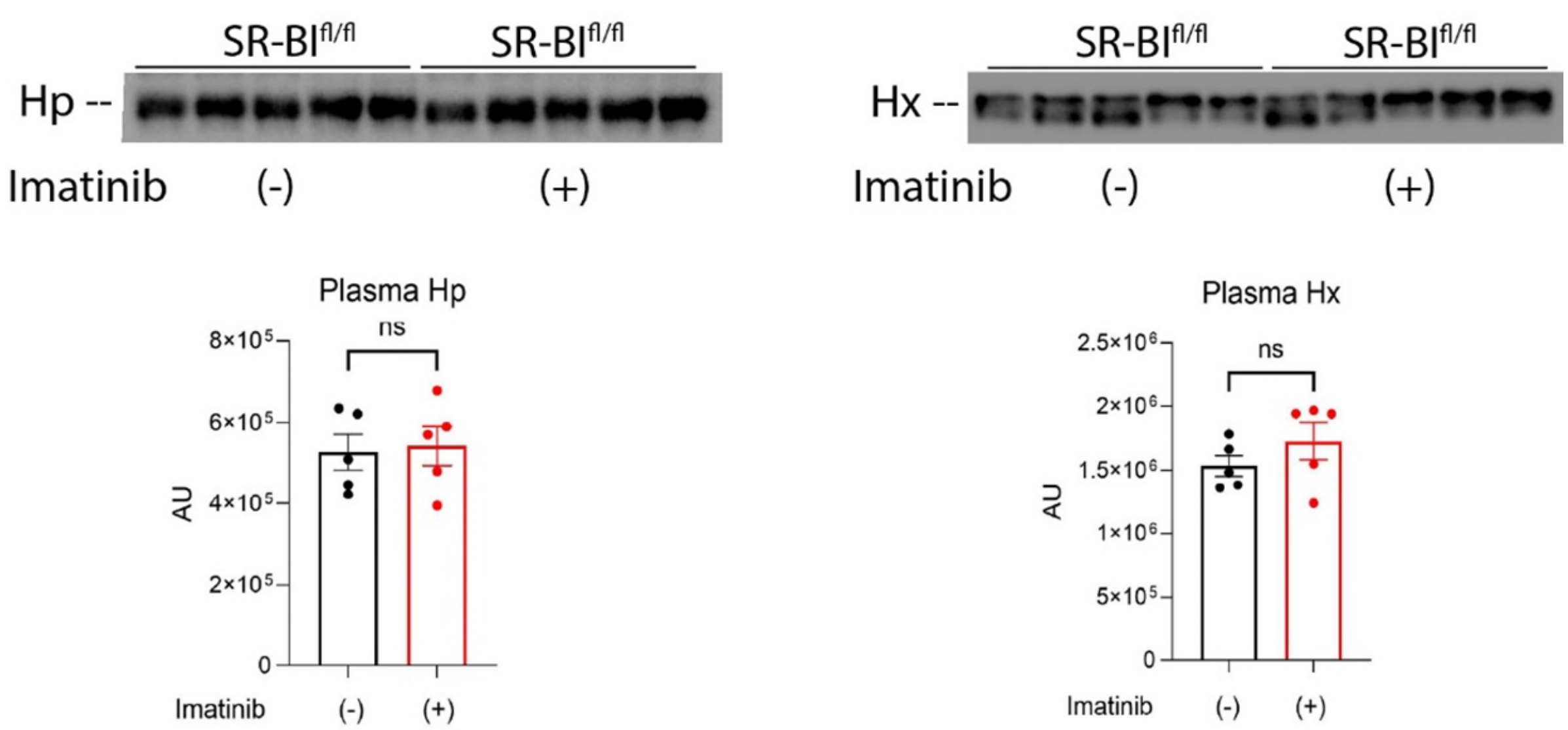

Figure 6. SR-BI$^{fl/fl}$ (n = 5) mice were treated with or without imatinib (50 mg/kg BW) for 3 days. 30 µg of plasma protein was loaded and examined for Hp and Hx via immunoblot. Significance was determined by Mann-Whitney test.

Next, leveraging a recently published transcriptomic dataset of SCD Townes mice liver, we analyzed normalized expression counts (via DESeq2) and heatmap profiles under baseline and heme-induced early crisis[14]. Among the hemolysis-scavengers genes examined (SR-BI, CD91, CD163, Hx, Hp), SR-BI displayed the most pronounced trend toward downregulation during crisis, accompanied by the lowest adjusted p-value (**RL Figure 7**). In contrast, canonical scavenger receptors remained comparatively stable. These transcriptomic findings reinforce the notion that hepatic SR-BI likely play a distinct and responsive role in heme clearance during acute hemolytic stress.

This downregulation trend of liver SR-BI seen in the SCD Townes mice during early heme crisis closely mirrors our observations in the wild-type mice. Our data showed that hepatic SR-BI had a significant downregulation following a heme crisis (**RL Figure 8**). Together, this data strongly suggests that SR-BI responds acutely to heme exposure, even if broader time-course comparisons dilute the signal. We added this new data to Manuscript Result section page 10, line 348 to 357 and supplemental figure S4.

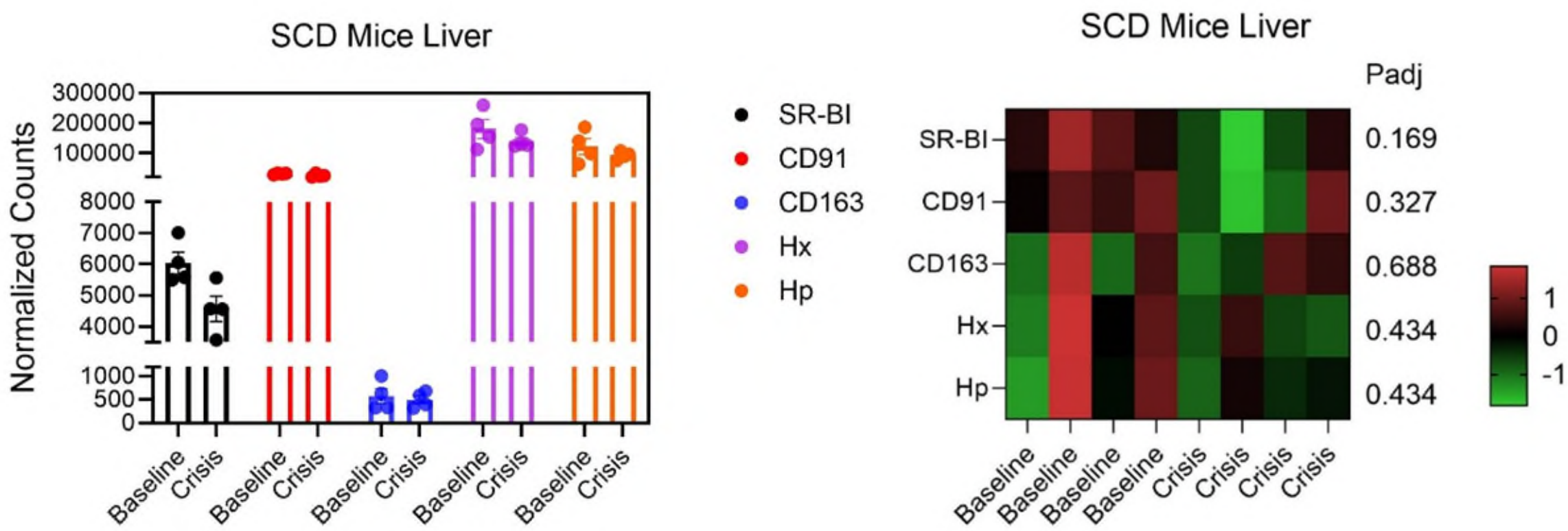

Figure 7. Normalized gene count (via DEseq2) and heatmap of hemolysis scavenger gene expression at the liver of SCD Townes mice (n=4).

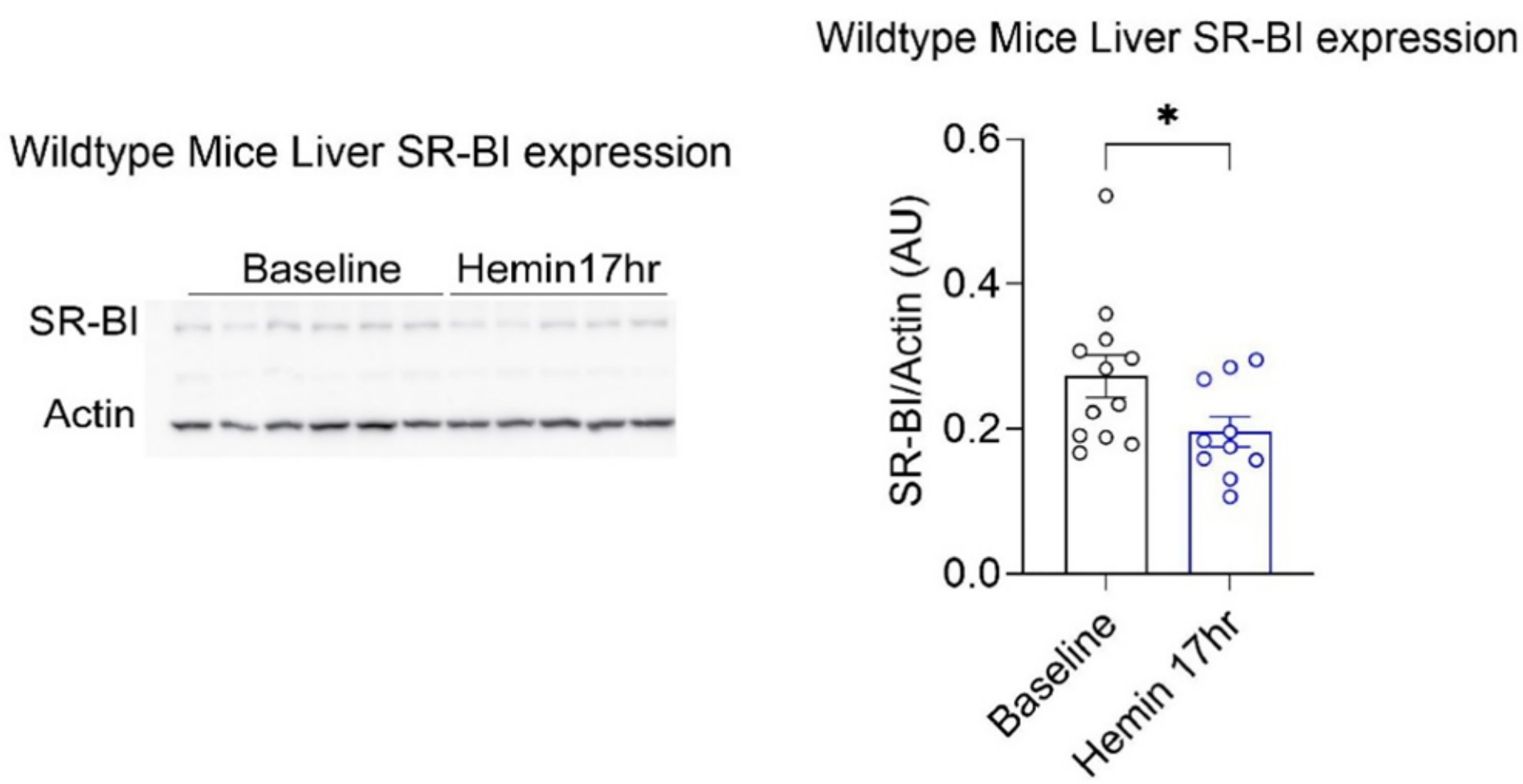

Figure 8. SR-BI$^{fl/fl}$ (n = 10-12) mice were injected with 40 µmol/kg BW of hemin (i.p.). Livers were collected at 17 hrs after hemin injection. 30 µg of liver protein was loaded and examined for SR-BI level via immunoblot. * P < 0.05; Significance was determined by Mann-Whitney test.

Lastly, we agree with the reviewer that genetic loss of Hp and Hx would further clarify the contribution of SR-BI to pathological heme clearance. However, generating triple-knockout mice (SR-BI, Hp, and Hx) is technically challenging and raises additional concerns about developmental compensation that affects baseline phenotypes (I.e. HpHx DKO mice develop splenomegaly at baseline).

To avoid these confounders, we adopted the established protocol developed by Dr. Pawlinski's group, in which endogenous Hp and Hx are acutely depleted prior to heme challenge[15]. We followed this approach in our study and subsequently performed heme-clearance measurements. This strategy allows us to directly test heme handling in the absence of Hb/heme scavenger proteins without the complications introduced by multi-allelic germline knockouts.

Reviewer 2 (Remarks to the Author):

Ito and colleagues identify the scavenger receptor BI (SR-BI) as a mediator of free heme clearance. The findings are novel and of clinical relevance, with important implications for disorders associated with hemolysis. This reviewer has a few suggestions for additional experiment for the author's consideration. Additionally, the data analyses should be reviewed and likely revised as to ensure appropriate statistical analysis of the data

1. The authors point out that the SR-BI binds to heme with low affinity with a Kd at 18.2 µM determined with SPR. The authors should provide addition information from the kinetic traces. Did the authors determine association and dissociation rates?

--- Yes, in addition to the equilibrium Kd derived from the steady-state SPR binding isotherm, we also extracted kinetic parameters from the association and dissociation phases of the sensorgrams. At 30 µL/min, with association for 90 s, and dissociation for 210 s, the association rate constant was $Ka = 1.10 \times 10^3\ M^{-1}\cdot s^{-1}$, and the dissociation rate constant was $Kd = 2.00 \times 10^{-2}\ s^{-1}$. We have revised Results section Figure 1 legend Page 4 line 158 to 160 to include this important information.

2. Apart from computational modeling, the authors should consider confirming the heme/SR-BI interaction using other methods – NMR, cryo-EM.

--- We thank the reviewer for this thoughtful suggestion. As a laboratory with a longstanding focus on SR-BI biology, we have proactively explored structural approaches to directly interrogate the SR-BI–heme interaction, including consultations and attempted collaborations with structural biologists specializing in membrane proteins. As an initial and essential step, we undertook extensive efforts to express and isolate SR-BI from mammalian expression systems, including CHO cells. While SR-BI expression in these cells was robust, repeated and systematic optimization attempts failed to yield sufficient quantities of stable, homogeneous, soluble SR-BI. As a result, we were unable to generate material suitable for X-ray crystallography or cryo-EM analysis. Taken together with the well-recognized challenges associated with applying high-resolution structural methods to large, glycosylated, multi-pass membrane receptors, these experimental constraints led us to conclude that NMR, cryo-EM, or crystallographic approaches are not presently feasible or likely to produce interpretable structural information for the SR-BI–heme interaction.

To date, high-resolution NMR work on SR-BI has been limited to the C-terminal transmembrane fragment (residues 405–475), whereas no full-length SR-BI structure has been determined by NMR[16]. SR-BI is a large, multi-domain transmembrane receptor, which makes solution NMR of the intact receptor in a native-like membrane environment technically challenging and currently beyond what has been achieved for this protein[17]. Furthermore, heme is a small paramagnetic ligand, which creates a ~20 Å 'blind sphere' around the binding site due to paramagnetic relaxation enhancement[18]. This obliterates signals needed for site-specific assignment or structural characterization of the SR-BI–heme complex. Given these limitations, the NMR approach was dismissed as a complementary approach. High-resolution structural work on SR-BI homologues has been limited to soluble ectodomains[19]. Even in these optimized systems, small ligands were not directly visualized, and ligand interactions were inferred through modeling and confirmed via functional assays. This underscores the difficulty of using structural methods such as X-ray crystallography or cryo-EM to resolve a small cofactor like heme within the flexible, membrane-embedded regions of SR-BI.

Given these documented limitations, we determined that an integrated approach—combining computational modeling with functional uptake assays, in vivo clearance experiment, bioinformatics approach —would be the most rigorous and informative strategy for defining the heme–SR-BI interaction in the present study. We have added clarifying text in the Supplemental data Page 1 to reflect this rationale and to note that future structural studies, once technically feasible, would be valuable for further refinement of the binding interface.

3. Additionally, kinetic experiments using receptor mutants of predicted binding sites could strengthen the results.

--- Following your suggestion, we examined the SR-BI C323G mutant. Prior studies, including ours, have shown that this mutant is correctly localized to the cell membrane (**RL Figure 9**) [20,21], yet completely lacks the ability to uptake cholesterol esters from HDL. This loss of function suggests that the C323G substitution disrupts the receptor's lipid channel and therefore provides a useful model for verifying SR-BI–dependent heme uptake. To investigate how this mutation affects heme binding, firstly we performed silico docking simulations on the C323G variant (**RL Figure 10**). When mutated, the C323G mutation breaks the disulfide bond located at the CPC (Residues 321 to 323) motif, a critical recognition motif that is conserved across the CD36 family of proteins, this breakage leaves a free cysteine residue to form errant dimers with other mutated versions of SR-BI, leading to malformation of the dimer structure and loss of protein functionality[22-24]. Even when the correct dimer positioning is able to form, the breakage of the disulfide bond leads to a contraction of the binding site, causing the central proline of the CPC motif to descend down into the binding site that Hemin occupies, blocking the WT binding mode of Hemin. Residues Y194 and T333 further contract the SR-BI binding site, destroy the strong hydrogen bond with N330, clashing against the peripheral methyl and vinyl carbons of the protoporphyrin ring. Additionally, the energy minimization of the C323G SR-BI dimer shows the K151 residue repositioning away from the center of the binding site, increasing the distance between it and Hemin by over 1.5 Å, reducing the favorable coulombic interactions between the two. Additionally, the cleavage of the C321–C323 disulfide bond results in an additional free thiol, decreasing the net positive charge of SR-BI and thus reducing the overall coulombic attraction between SR-BI and Hemin. These clashes within the binding site along with the malformed dimer creation, could result in impaired hemin transport by SR-BI.

Next, we validated these computational findings using ZnMP uptake assays (**RL Figure 11**). CHO cells expressing SR-BI C323G displayed markedly reduced ZnMP uptake, comparable to CHO-vector controls. We thank the reviewer for this valuable suggestion. Together, these new data further strengthen our conclusion that SR-BI mediates cellular heme uptake. We also revised our discussion in light of this new data. Result Page 3 line 109 to 121 and Supplemental data Page 1.

[editorial note: third party material redacted]

Figure 9 is adopted from Guo et al. *Journal of Lipid Research*. 2011;52:2272

Figure 9. Subcellular location of C323G mutant SR-BI. **A:** Detection of cell surface SR-BI expression by biotin labeling assay. CHO cells expressing vector, wild-type, or C323G mutant SR-BI were labeled with biotin. Biotinylated proteins were isolated with an avidin affinity column and analyzed by Western blot. **B:** Deglycosylation of wild-type and C323G mutant SR-BI. The CHO cells were lysed, treated with a mixture of enzymes to remove saccharide from protein, and then analyzed by Western blot. **C:** Subcellular targeting of SR-BI. Subcellular fractions were isolated from the CHO cells and analyzed by Western blot. Postnuclear supernatant (PNS), cytosol (CYT), intracellular membrane (IM), plasma membrane (PM), and caveolae membrane (CM) fractions.

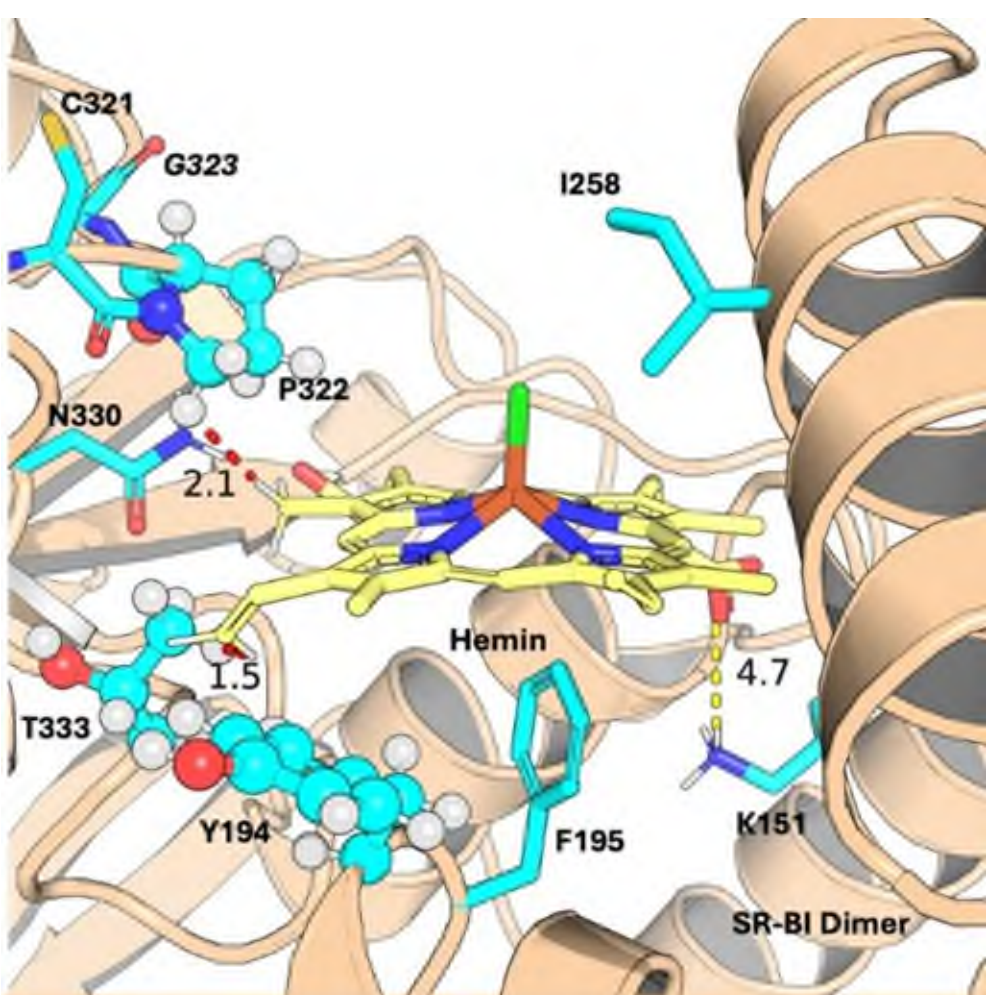

Figure 10. Binding modes of hemin and ZnMP with SR-BIC323G predicted using an energy-minimization workflow implemented in AutoDock Vina.

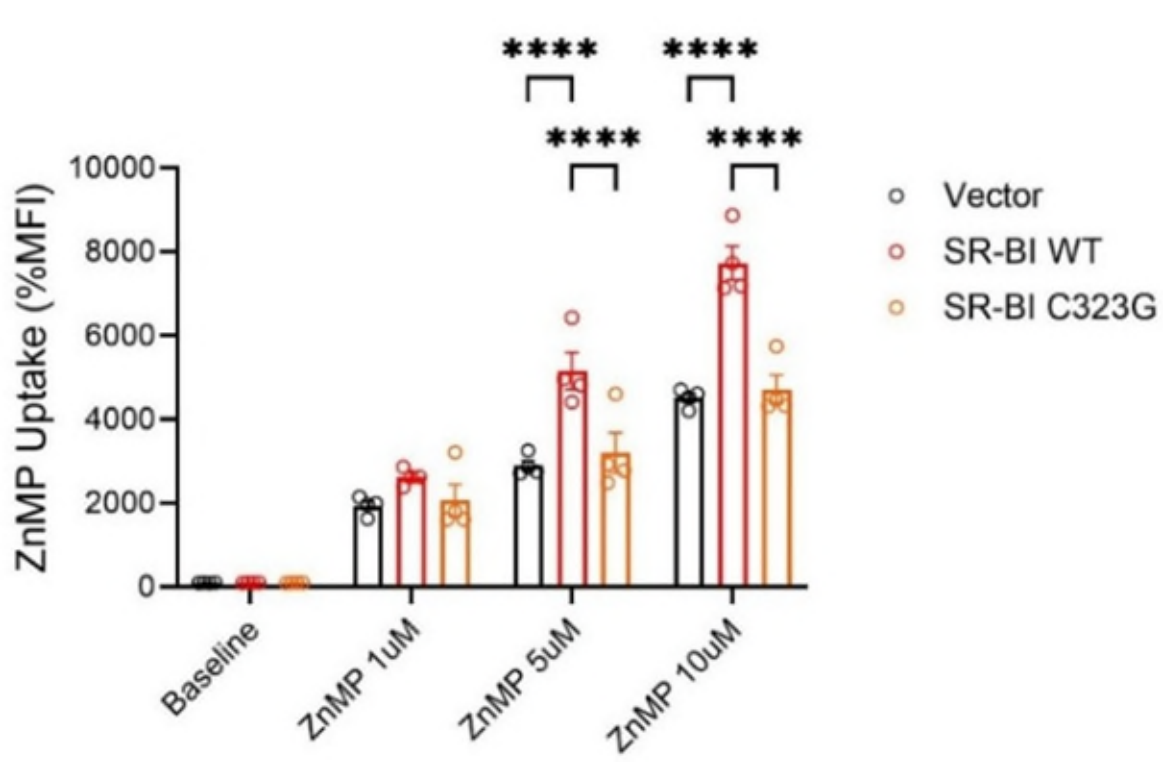

Figure 11. Flow cytometry analysis of ZnMP uptake in CHO cells expressing vector, SR-BI WT and SR-BI C323G. **** $P < 0.0001$; Significance was determined by two-way ANOVA.

4. Did the authors conduct SPR with reversed orientation? Different immobilization levels? Those experiment could improve the interpretation of the results and shed light on how such low affinity interaction yield the physiologic responses reported.

--- We thank the reviewer for this thoughtful suggestion. SPR measurements were conducted by a commercial service provider (Creative Biolabs) with SR-BI immobilized on the sensor chip. We agree that reversed-orientation SPR and testing different immobilization levels could provide additional mechanistic insight. However, as discussed above, despite extensive and repeated efforts, we were unable to obtain enough quantities of stable, homogeneous, soluble SR-BI to support alternative experimental configurations.

Importantly, the SPR measurements are well validated by multiple independent functional assays. The measured equilibrium dissociation constant (KD ≈ 18.2 μM) is internally consistent with our cellular uptake and HO-1 induction assays, which demonstrated robust SR-BI–dependent activity at ligand concentrations within or below this range. These data indicate that SR-BI–mediated hemin uptake and signaling occur efficiently within the concentration window predicted by the SPR measurements. Furthermore, our in vivo hemin clearance experiments showed rapid, SR-BI–dependent clearance, further supporting high functional efficiency despite modest binding affinity.

Notably, a micromolar affinity is fully compatible with physiological relevance in the context of hemolytic disease. In conditions such as sickle cell disease crisis, circulating free heme levels can rise well above the measured KD, making it entirely plausible for a low-affinity, high-capacity interaction to drive the biological responses observed.

Taken together, the convergent evidence from SPR measurements, cellular functional assays, and in vivo clearance studies supports the physiological relevance of SR-BI–heme interactions as presented in this study.

5. The experiments shown in Figure S1 examining the effect of HDL on ZnMP uptake should include a dose response both of HDL and ZnMP. A dose-response experiment would greatly add to the understanding of how do already known SR-BI ligands affect heme binding.

--- We thank the reviewer for this helpful suggestion. Following your comment, we performed a dose-response analysis using HDL at 10, 50, 100 μg/mL to test how a known SR-BI ligand modulates heme analog uptake. The new data (**RL Figure 12**) show a dose-dependent inhibition of ZnMP uptake by HDL. While vector cells show minimal change at a low HDL level, SR-BI cells exhibit progressive suppression of

ZnMP uptake with increasing HDL concentrations. We have added this data to Results Section Page 4 Line 148 to 152 and Supplemental Figure 1. These results strengthen the conclusion that HDL competes with heme analog binding/transport through SR-BI.

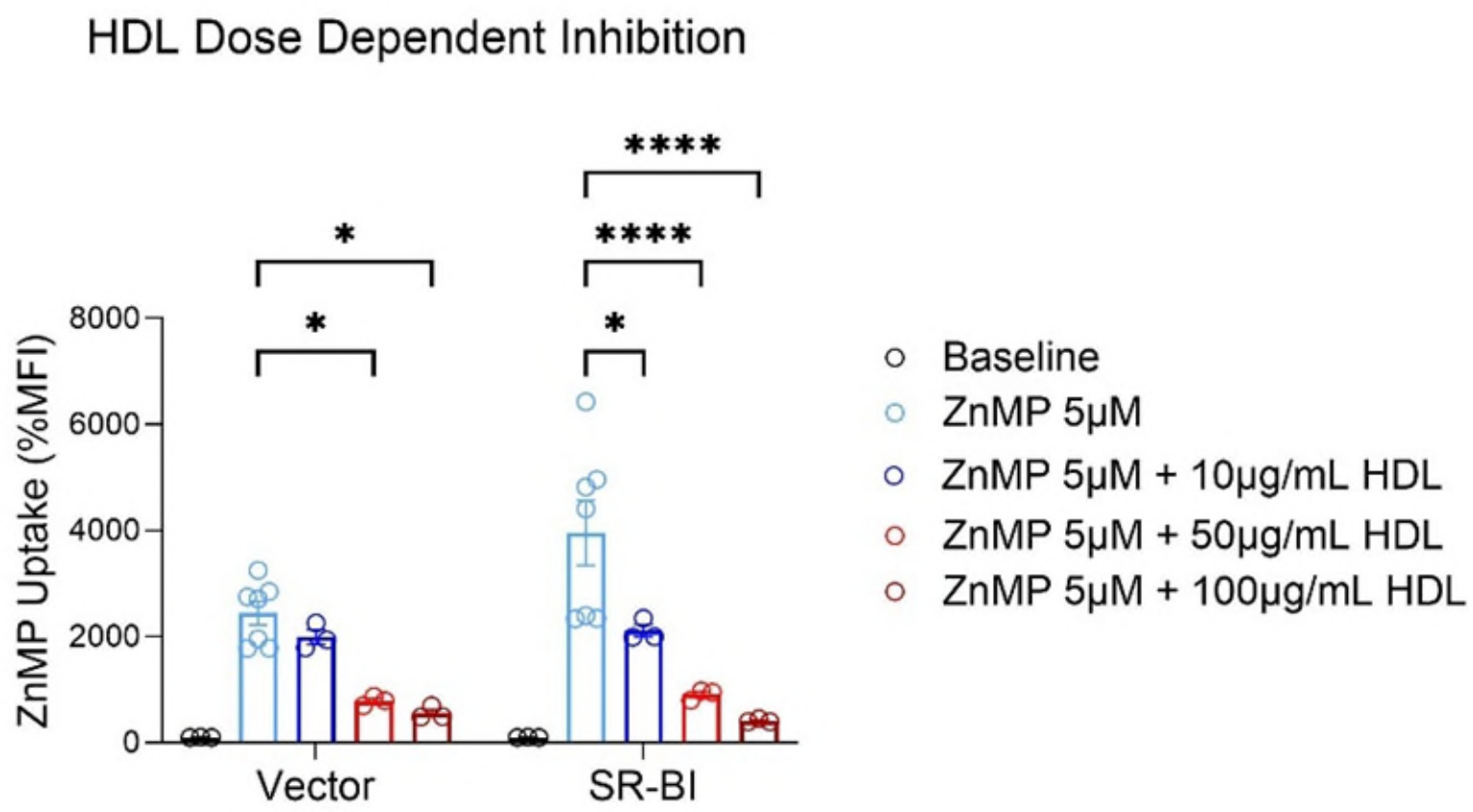

Figure 12. Flow cytometry analysis of ZnMP (5 µM) uptake in CHO cells expressing vector, or SR-BI in the presence of 10, 50, 100 µg/ml high-density lipoprotein (HDL). * P < 0.05; **** P < 0.0001; Significances were determined by two-way ANOVA.

6. Figure 2 panels b and c – note that blood is collected from the mice 4 times over a period of 60 min. How much is drawn each time? What are the implications of phlebotomy on plasma hemin concentration and free hemin clearance? Was the volume replaced? In those figures what analyses were performed – the figure legend indicates that unpaired Mann-Whitney test for hemin clearance. This is not appropriate as there are two factors that might affect hemin levels: the model (SR-BIfl/fl and Albcre SR-BIfl/fl) and time – a two-way repeated measures ANOVA is more appropriate rather than unpaired Mann-Whitney test for hemin clearance as the legend intimates.

--- We thank the reviewer for this thoughtful comment regarding statistical and methodological approach.

Regarding phlebotomy: Each blood draw consisted of 30 µL, representing approximately <2% of total blood volume, which is well within established humane endpoints for serial sampling in mice. At these volumes, phlebotomy-induced changes to plasma volume or hemin concentration are negligible and would not significantly impact hemin clearance kinetics. Blood volume was not replaced, as the small volume removed does not require replacement per institutional IACUC guidelines.

Regarding statistical analysis: Following your suggestion, we revised the statistical analysis with 2-way ANOVA and updated the figure (**RL Figure 13**) and in Manuscript Results Figure 2 Page 7.

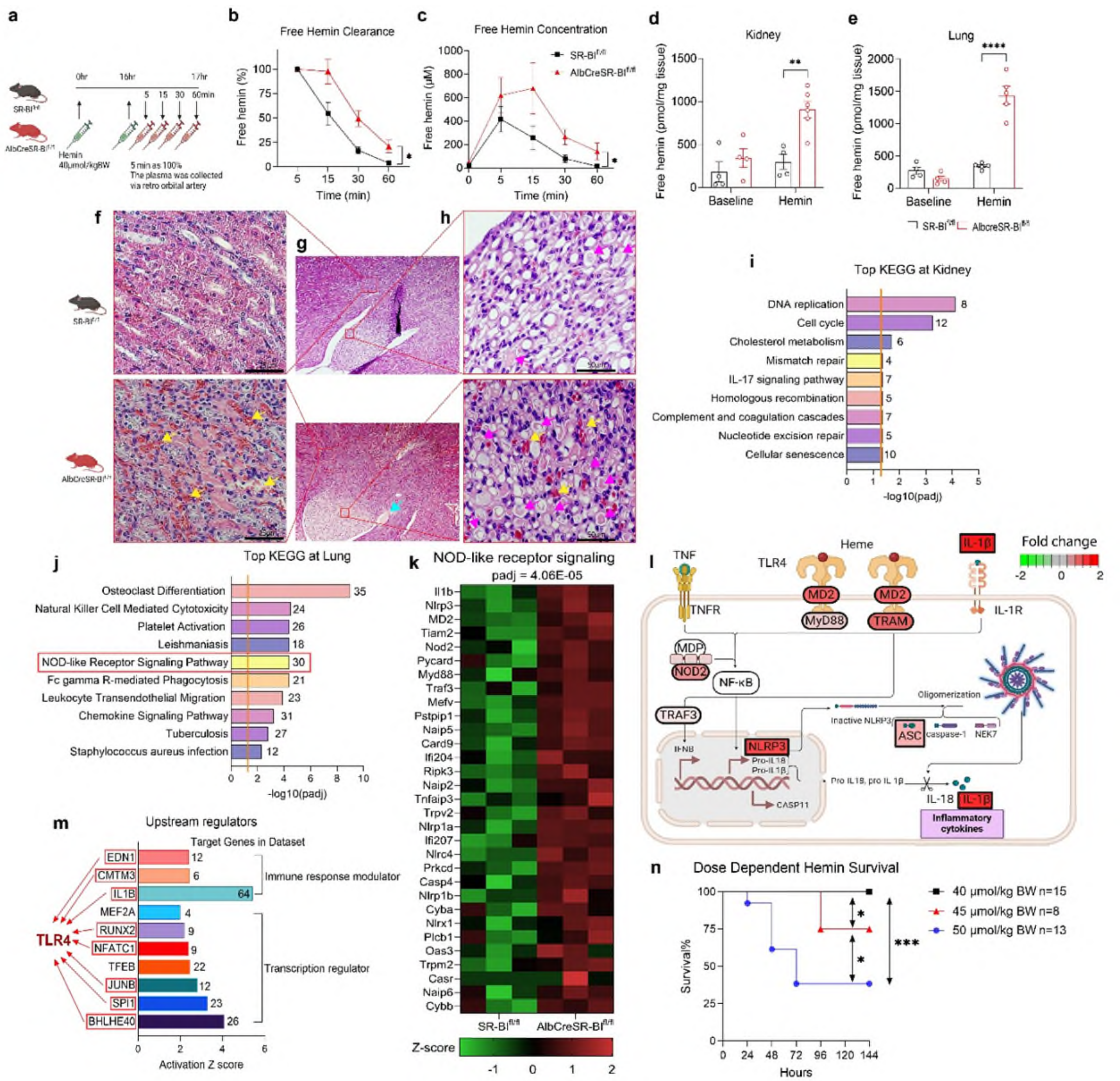

Figure 13. Hepatic SR-BI promotes heme clearance and alleviates heme-induced signaling.

(**a**) Experimental design of hemin clearance. SR-BI$^{fl/fl}$ and AlbcreSR-BI$^{fl/fl}$ (n = 4-6) mice were injected with 40 µmol/kg BW of hemin (i.p.). Sixteen hours later, the same dose of hemin was injected (i.v., green syringe) and retroorbital blood was collected at indicated time points for measurement of free heme (red syringe). (**b-c**) Free hemin clearance. The plasma free hemin levels at 5 mins were set as 100% of injected hemin. (**d-e**) Free hemin levels in the kidney and lung tissue lysates were measured one hour post the 2nd hemin injection. (**f-h**) Representative histological images of kidney sections from SR-BI$^{fl/fl}$ and AlbcreSR-BI$^{fl/fl}$ 14 hours post the 2nd hemin injection. (**i-j**) Top KEGG enrichment pathway at kidney and lung. Number next to each bar represents the number of DEGs in that pathway (**k**) Heatmap represents DEGs in NOD-like receptor

signaling KEGG pathway. (**l**) Illustrative cartoon of TLR4 and inflammasome activations. (**m**) IPA-predicted upstream regulators. The number within each bar describes target genes from dataset. Activation Z-score ≥ 2 indicates significance. (**n**) SR-BI$^{fl/fl}$ mice were intraperitoneally injected with 5 shots of hemin of various doses. Survival were monitored for 144 hours and analyzed by Log-Rank test; Significances were determined by mixed-effects linear regression and two-way ANOVA. * $P < 0.05$; ** $P < 0.01$; **** $P < 0.0001$.

[editorial note: panels a and f created in BioRender. Xue, J. (2026) https://BioRender.com/dka8qi9. Panel L created in BioRender. Xue, J. (2026) https://BioRender.com/a2sck22.]

7. Did the authors measure free hemin in the liver in the experiments described in Figure 2d and 2e?

--- Following your suggestion, we measured free hemin in the liver (**RL Figure 14**). We did not detect a significant difference in hepatic free hemin between control and liver-specific SR-BI knockout mice. Our interpretation is that, although loss of hepatic SR-BI is expected to reduce hepatocellular heme uptake, markedly elevated circulating free hemin likely permits passive diffusion into liver tissue, thereby masking differences in total hepatic hemin content. In contrast, free hemin was significantly increased in the kidney and lung—organs in which SR-BI expression is preserved. These data have been added to Supplemental Figure 2 and the Results section (page 5, lines 183–187).

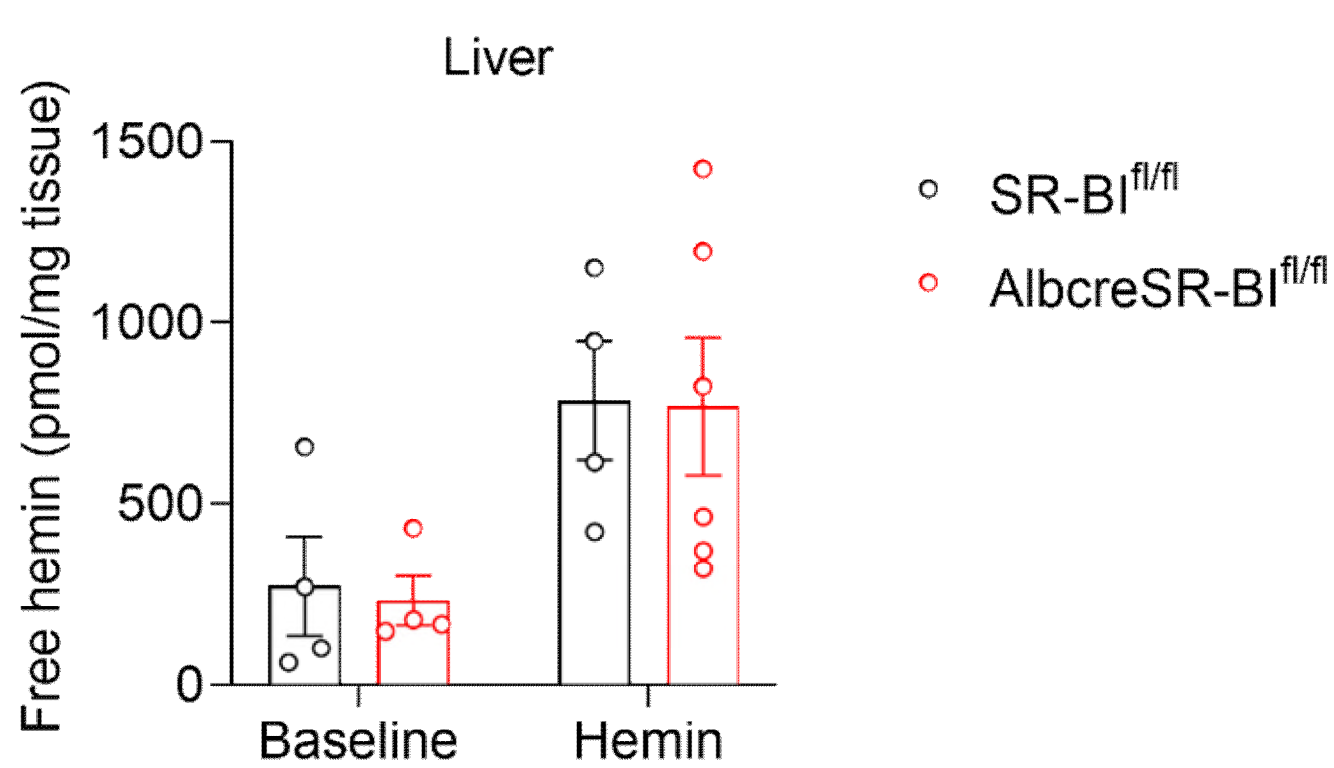

Figure 14. Free hemin levels in the liver tissue lysates were measured one hour post the 2nd hemin injection from the free hemin clearance study.

8. What hepatic and metabolic alterations do Albcre SR-BIfl/fl mice present? If any could those interfere on the animals' susceptibility to hemin?

--- Following this suggestion, we conducted a comprehensive baseline biochemical and metabolic assessment of wild-type and AlbCreSR-BI$^{fl/f}$ mice. Analysis of serum biochemistry and organ function markers revealed no significant differences between genotypes, indicating comparable hepatic and metabolic status at baseline (**RL**

Figure 15; Fig. S3i–v). In addition, we measured baseline parameters related to red blood cell and heme homeostasis, including free hemin, red blood cell count (RBC), hemoglobin (Hb), hematocrit (HCT), reticulocyte percentage (RET), and spleen-to-body-weight ratio (**Fig. 3**). No significant differences were observed between wild-type and AlbCreSR-BI$^{fl/f}$ mice across these measures. Together, these results indicate that loss of hepatic SR-BI does not result in baseline hepatic, metabolic, or hematologic abnormalities that could confound susceptibility to hemin challenge. These data have been added to **Supplementary Figures S3i–v** and incorporated into the Results section (page 8, lines 285–289).

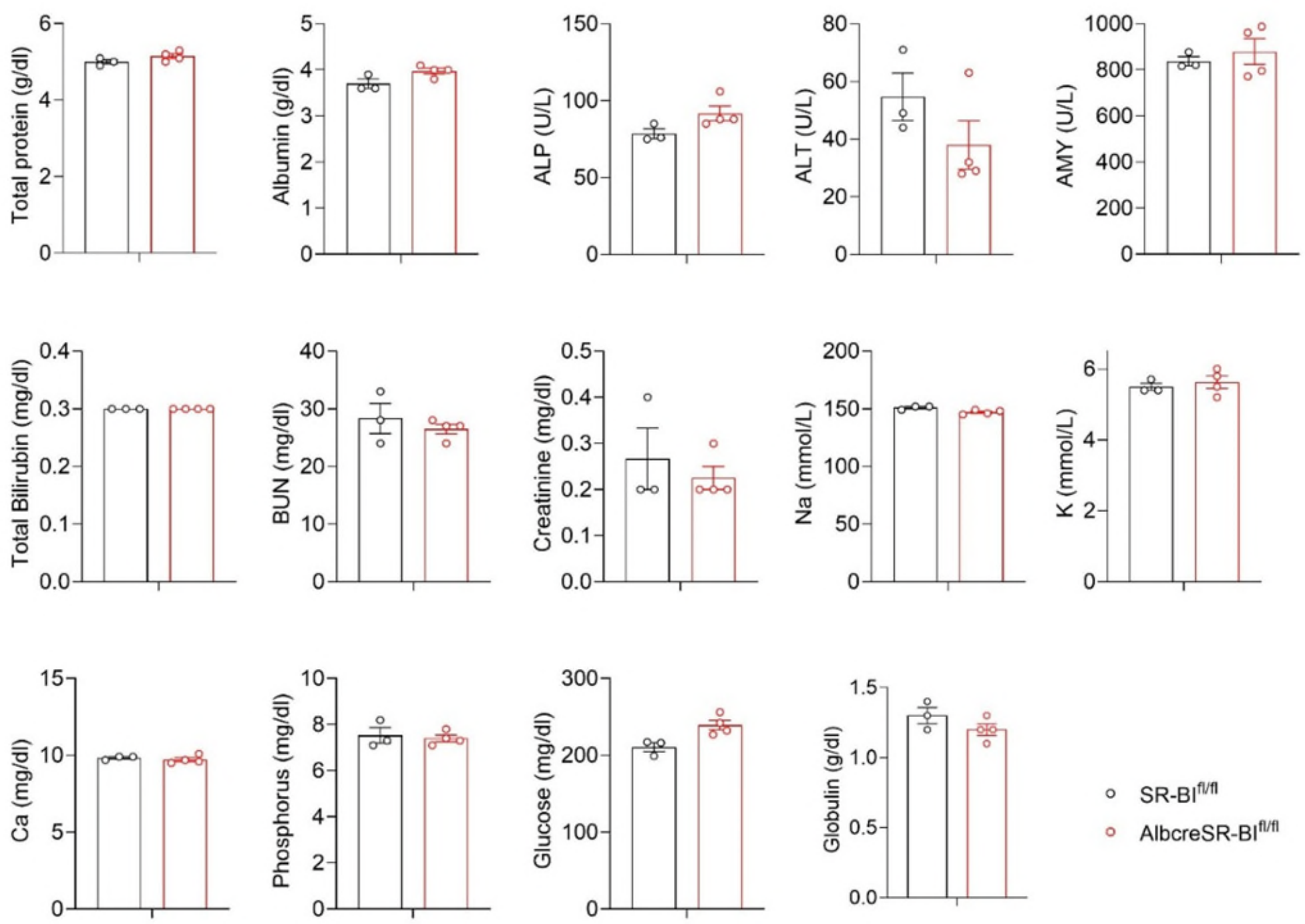

Figure 15. Biochemistry panel of SR-BI$^{fl/fl}$ (n=3) and AlbcreSR-BI$^{fl/fl}$ (n=4) at baseline. Means ± SEM are plotted. Significances were determined by Mann-Whitney test.

9. In the model of sickle cell disease, non-SCD littermates may not be the ideal controls as these animals express mouse hemoglobin. The authors should use the HbAA – BERK mice that akin to the SCD-BERK do not express any mouse hemoglobin genes but human hemoglobin A.

--- We thank the reviewer for this important point. In our study, the non-SCD littermates used as controls are indeed HbAA-BERK, which express human hemoglobin A but not hemoglobin B^{sickle}. This control strategy follows established precedents in the field. For example, Cain et al. stated in their method section that similar littermate mixed genetic strain background mice expressing normal human hemoglobin, HbAA-BERK were used as control for HbSS-BERK mice, from the same breeding[25]. We updated Figure 4 in the Results section on page 11 which now labels non-SCD littermates as HbAA-BERK.

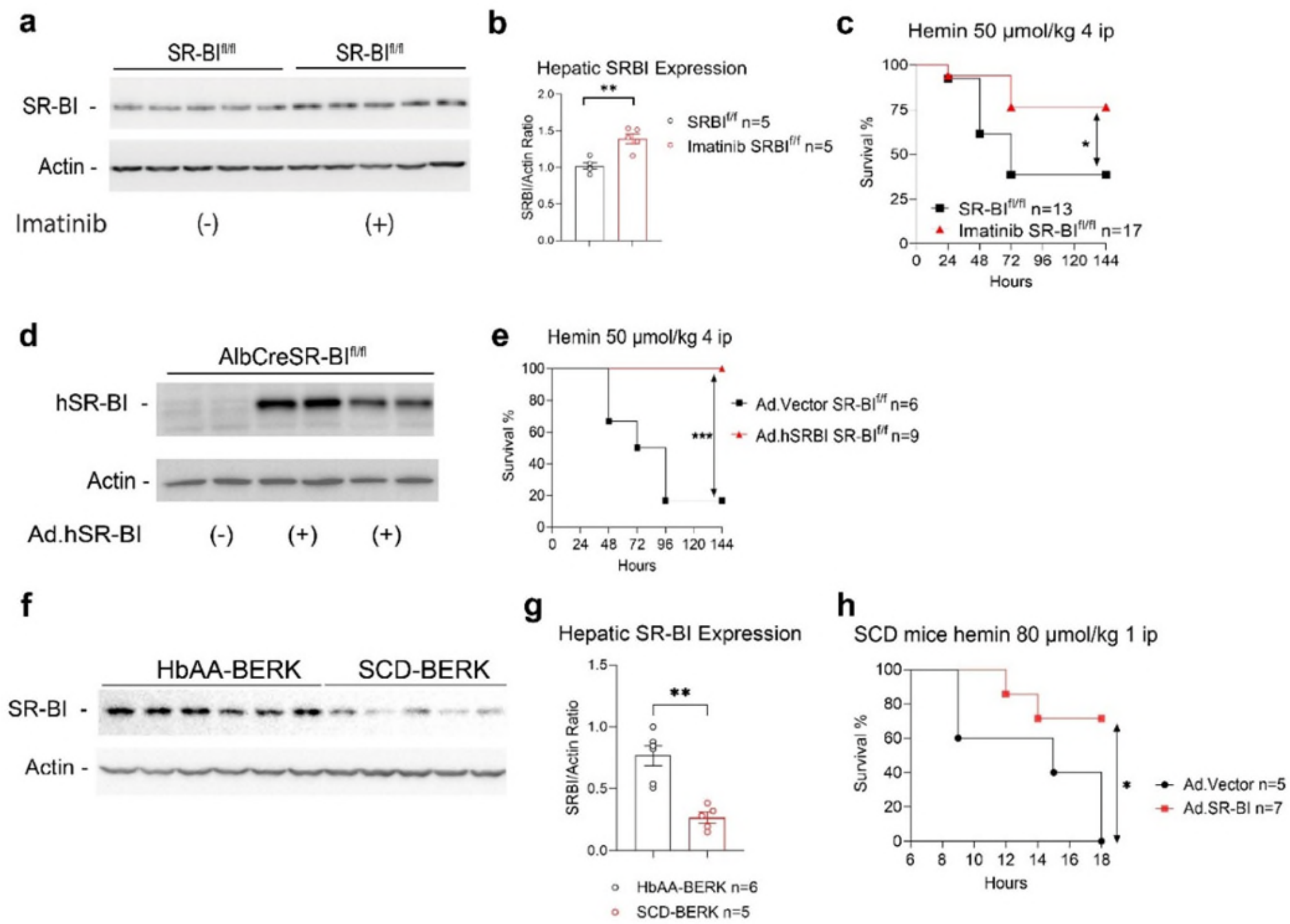

Figure 16. Upregulation of hepatic SRBI protects against heme-induced lethality.

(**a-b**) Hepatic SR-BI expression in SR-BI$^{fl/fl}$ mice treated with/without imatinib (50 mg/kg BW) for 3 days, followed by daily hemin injection of 40 µmol/kg for 5 days. (**c**) Survival analysis of SR-BI$^{fl/fl}$ mice treated with/without imatinib for 3 days, followed by daily hemin injection. (**d**) Hepatic SR-BI expression in AlbcreSR-BI$^{fl/fl}$ mice treated with adenovirus expressing human SR-BI (Ad.hSR-BI) or vector (Ad.vector) for 3 days. (**e**) Survival analysis of SR-BI$^{fl/fl}$ mice treated with Ad.hSR-BI or Ad.vector for 3 days, followed by daily hemin injection. (**f-g**) Hepatic SR-BI expression in Non-SCD mice (Hba$^{-/-}$Hbb$^{+/-}$Tg$^{+/-}$ n=6) and SCD-BERK littermates (Hba$^{-/-}$Hbb$^{-/-}$Tg$^{+/-}$) after daily hemin injection of 45 µmol/kg for 5 days. (**h**) Survival analysis of SCD-BERK mice treated with Ad.SR-BI or Ad.vector for 3 days, followed by hemin injection. Data are shown as means ± SEM. * *P* < 0.05; ** *P* < 0.01; *** *P* < 0.001, determined by Student's t test. Survival curves were analyzed by the Log-Rank test.

10. Imatinib has been shown to have beneficial effects in SCD mice. The authors should consider an experiment evaluating the effect of imatinib on SR-BI expression and mortality in hemin -challenged SCD mice. This would add to the translational potential of the study.

--- We thank the reviewer for this thoughtful suggestion. We agree that evaluating the relationship between imatinib, SR-BI, and heme toxicity in a sickle cell disease (SCD) background is important for translational relevance. In response, we performed additional experiments in SCD-Berk mice and confirmed that imatinib treatment upregulates hepatic SR-BI expression in this chronic hemolytic setting (**RL Figure 17**).

These new data are now included in the revised manuscript (**Supplemental Fig. S4**) and the Results section page 10, line 343- 347.

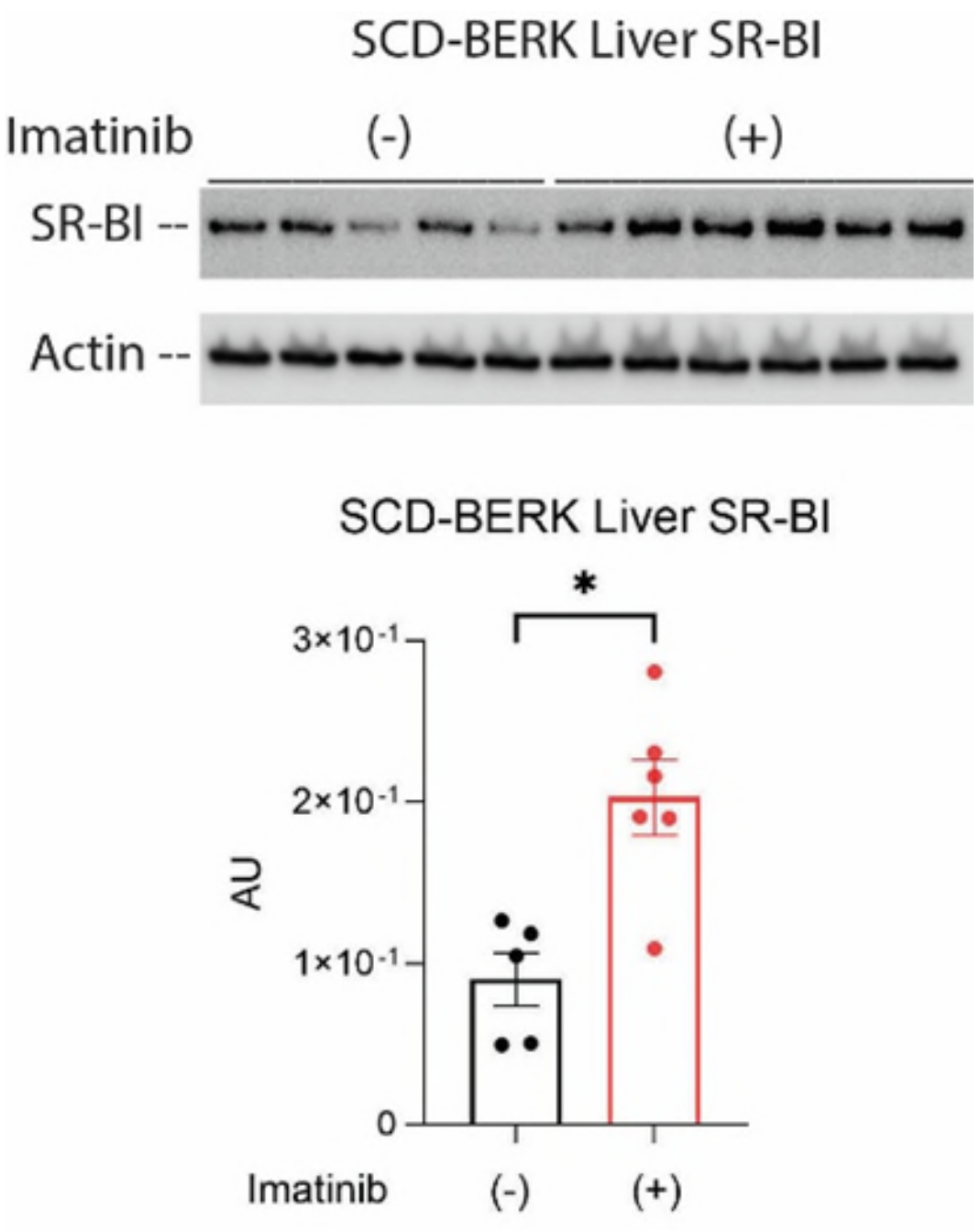

Figure 17. SCD-BERK mice (n=5-6) were treated with or without imatinib (50 mg/kg BW) for 3 days. 30 µg of liver protein was loaded and examined for SR-BI level via immunoblot. Data are shown as means ± SEM. * $P < 0.05$; determined by Mann-Whitney test.

Regarding the suggested mortality experiment using imatinib, prior studies have already demonstrated that imatinib confers broad protective effects in SCD mouse models across multiple pathophysiological domains[26,27]. Given these prior studies, an imatinib-plus-hemin mortality experiment in SCD mice cannot safely conclude that it is a SR-BI dependent mechanism that offered the protection. Because our primary goal was to determine whether SR-BI itself is a mechanistic determinant of protection from heme toxicity in the SCD context, we instead employed a gain-of-function strategy using adenoviral overexpression of SR-BI (Ad.hSR-BI) in SCD Berkeley mice. This approach allowed us to directly interrogate the role of SR-BI in heme tolerance within the SCD background while avoiding confounding effects of imatinib. Consistent with our findings in wild-type mice, hepatic SR-BI overexpression in SCD mice conferred significant protection against heme-induced lethality, demonstrating that SR-BI represents a key protective pathway against heme toxicity in SCD.

Together with our loss-of-function data showing that imatinib fails to provide significant protection in AlbCreSR-BI$^{fl/fl}$ mice, these results establish a mechanistically coherent framework in which SR-BI is necessary for imatinib-mediated protection and sufficient to confer protection when directly overexpressed in SCD mice.

We revised the Discussion section Page 13, line 422 to 434, clarifying that our study focuses on defining SR-BI as a mechanistic mediator of heme detoxification and as one pathway through which imatinib may exert beneficial effects in SCD. Long-term therapeutic studies in genetic SCD models remain an important future direction.

11. The experiments involving administration of Ad.Vector and AdhSRBI should include control mice (SR-BIfl/fl, in figure 4e and non-SCD and HbAA-BERK controls in figure 4H). How do we know there is no untoward effect of Ad.Vector injections.

--- Following your suggestion, we added side-by-side survival comparison of SR-BI$^{fl/fl}$ mice with or without receiving adenovirus vector (**RL Figure 18**). Under the same hemin treatment, survival with or without Ad.Vector is closely matched, ruling out the potential untoward effect of adenovirus. We have added this comparison to Supplemental Figure 4 and the Results Section Page 10 and line 327.

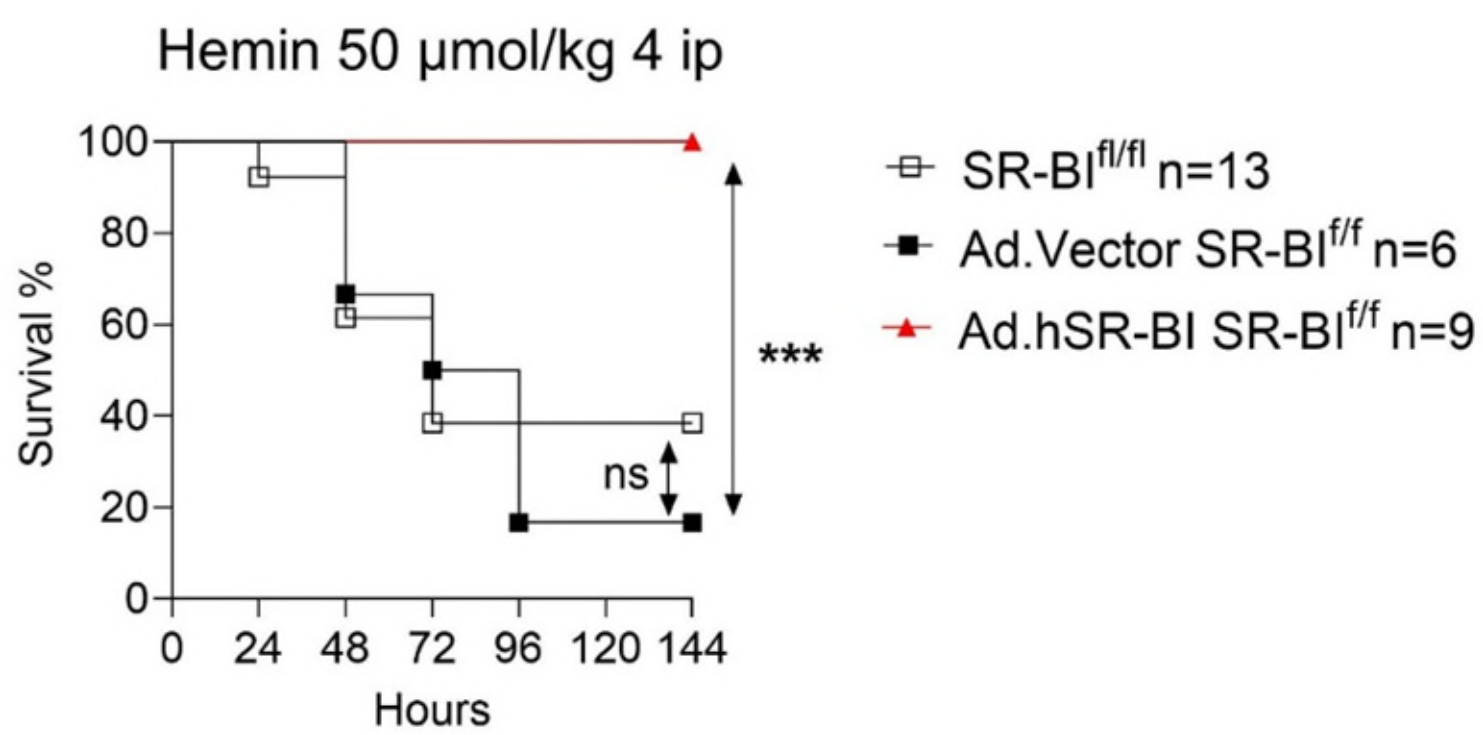

Figure 18. Survival analysis of SR-BI$^{fl/fl}$ mice treat with no adenovirus, Ad.hSR-BI or Ad.vector for 3 days, followed by daily hemin injection. *** $P < 0.001$; Survival curves were analyzed by the Log-Rank test.

12. Please revise the statistical analysis. Longitudinal data with repeated measures should be accounted for in the analysis. Please show the data points for each mouse in repeated measures experiments – Figure 1n-p; Figure 2 B and 2C

--- Following your comment, we made new graphs with the individual data points (**RL Figure 19**) and included them at Supplemental Figure 1. We thank the reviewer for raising this point.

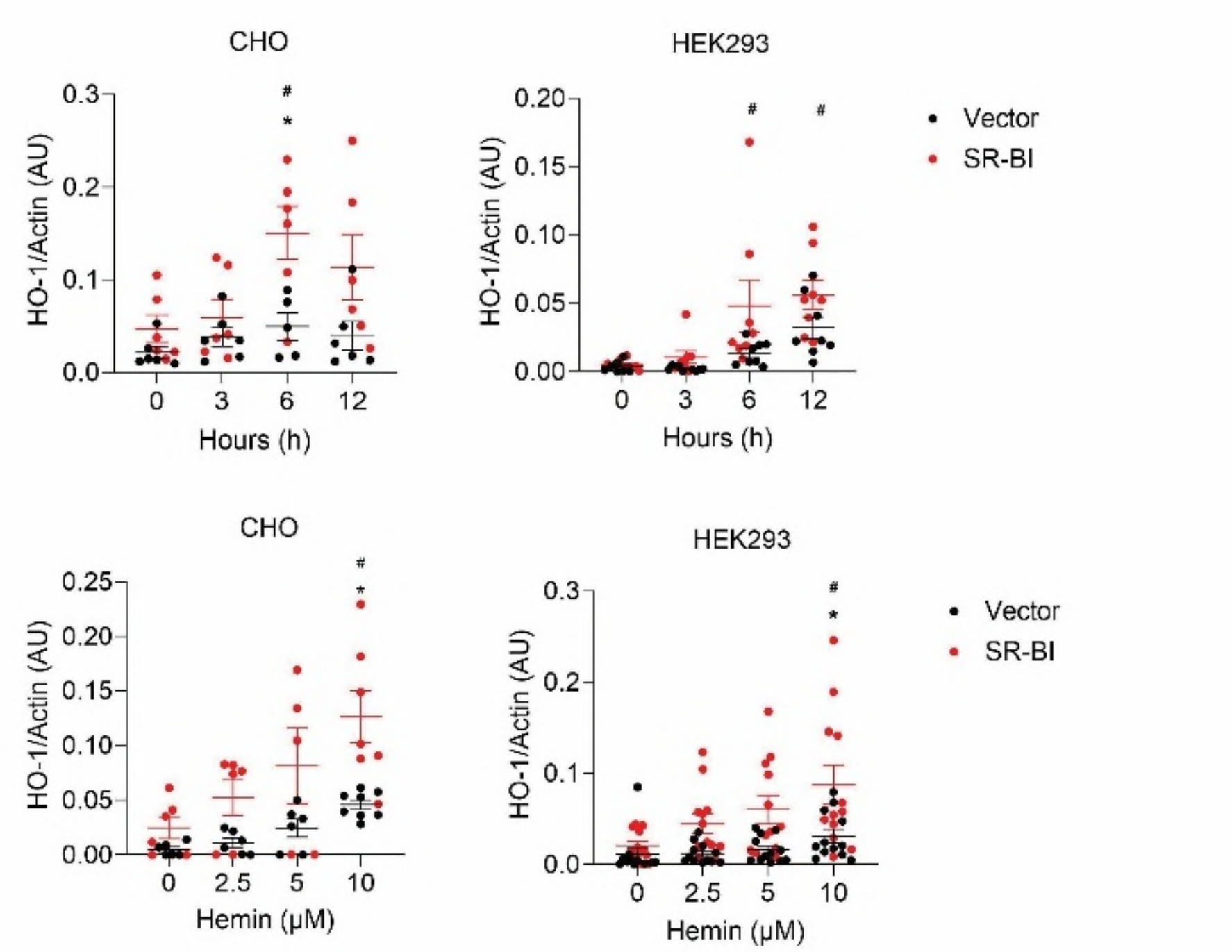

Figure 19. Individual data point for hemin time and dose-dependent HO-1 expression in Manuscript Figure 1 (m-p). The * denotes comparison with the vector control at the same timepoint, while # denotes comparison with baseline. Means ± SEM are plotted from three independent experiments. * P < 0.05; ** P < 0.01; *** P < 0.001; **** P < 0.0001; Significances were determined by two-way ANOVA.

Minor point.

1. Please provide spleen to body weight ratio rather than spleen weight alone as to control for differences in mouse sizes

--- The figure was updated to show spleen to body weight ratio (mg/g BW) (**RL Figure 20**). We revised Manuscript Figure 3.

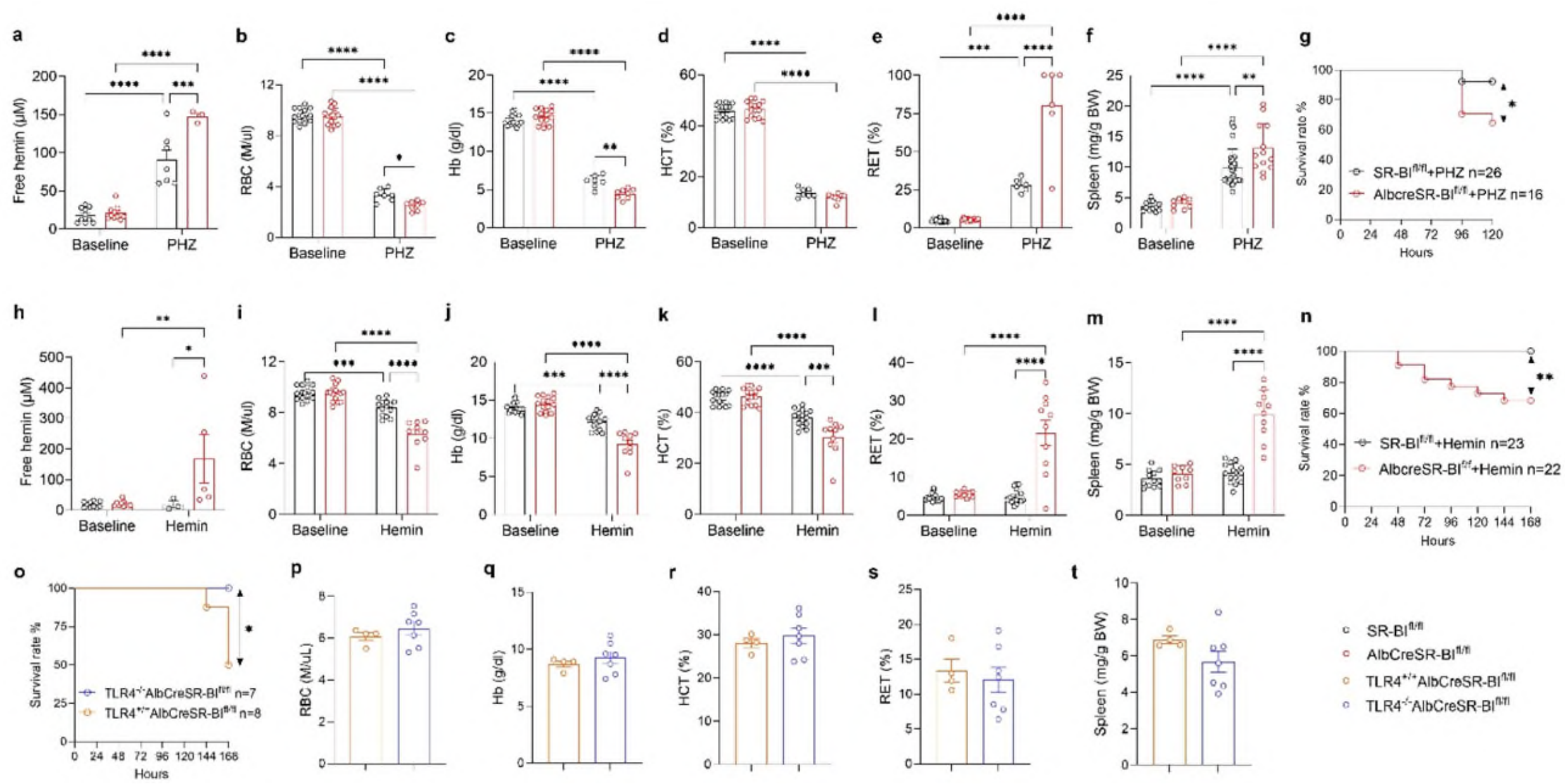

Figure 20. SR-BI protects against hemolysis- and heme-induced lethality.

SR-BI$^{fl/fl}$ and AlbcreSR-BI$^{fl/fl}$ mice were intraperitoneally injected with PHZ 30mg/kg BW (4 shots). (**a**) Free hemin levels were measured before and 72 hours after PHZ injections. (**b-e**) The complete blood counts including red blood cells (RBC), hemoglobin (Hb), hematocrit (HCT), and reticulocyte percentages (RET%) were measured at baseline and 120 hrs. SR-BI$^{fl/fl}$ (n= 7-15), AlbcreSR-BI$^{fl/fl}$ (n= 6-15). (**f**) Spleen weights, before and after PHZ injections. SR-BI$^{fl/fl}$ (n=9-30), AlbcreSR-BI$^{fl/fl}$ (n= 9-13). (**g**) Survival rates after PHZ injection. (**h**) SR-BI$^{fl/fl}$ (n=3-10), AlbcreSR-BI$^{fl/fl}$ (n=5-10) mice were intraperitoneally injected with hemin 40 µmol/kg BW (2 shots). Free hemin levels were measured before and 1hr after second hemin injections. (**i-l**) SR-BI$^{fl/fl}$ and AlbcreSR-BI$^{fl/fl}$ mice were intraperitoneally injected with hemin 40 µmol/kg BW (5 shots). The complete blood counts were measured at baseline and 168 hours. SR-BI$^{fl/fl}$ (n=15), AlbcreSR-BI$^{fl/fl}$ (n= 10-15). (**m**) Spleen weights, before and after hemin injections. SR-BI$^{fl/fl}$ (n=15), AlbcreSR-BI$^{fl/fl}$ (n= 9-10). (**n**) Survival rates after hemin injections. (**o**) Survival rates. TLR4$^{+/+}$AlbcreSR-BI$^{fl/fl}$ and TLR4$^{-/-}$AlbcreSR-BI$^{fl/fl}$ littermates were intraperitoneally injected with hemin 45 µmol/kg BW (5 shots). (**p-t**) CBC and spleen weights were obtained at 168 hours. Means ± SEM are plotted. * $P < 0.05$; ** $P < 0.01$; *** $P < 0.001$; **** $P < 0.0001$; Significances were determined by two-way ANOVA and Log-Rank test.

Reviewer #3 (Remarks to the Author):

Ito et al. describe the role scavenger receptor BI (SR-BI) in free heme clearance by the liver. This is demonstrated by addition of free heme in a mouse model lacking SR-BI, resulting in significant heme toxicity, and by upregulating SR-BI with imatinib. They next demonstrate that hepatic expression of SR-BI is reduced in a sickle mouse model with hepatopathy, and that adenoviral mediated upregulation of SR-BI reduced heme toxicity in the sickle mouse model,

Originality and significance: SR-BI may be a drug target to reduce heme toxicity in SCD and other hemolytic conditions. **This has been known for over a decade**. Others have shown that imatinib reduced lung, kidney, and liver injury in a sickle mouse model, and its impact on an enzyme involved in heme catabolism. This paper does provide some new information regarding the SR-BI mechanism

Data & Methodology: Currently, there is little novel information. The sickle mouse is treated with a heavy hand; there should not be a need to add lethal amounts of hemin to see an effect of adenovirus upregulation of SR-BI. The finding that SR-BI is downregulated in SCD, a disease model that needs to clear heme, is interesting but not explored further.

Suggested improvements: This paper would be improved by more translational work; the upregulation of SR-BI by adenovirus in the sickle mouse model is a good proof of principle, but a drug study, particularly one with a record of use in humans, would bring this close to human use. RNASeq would be more informative in a disease model rather than a mouse model lacking SR-BI

Clarity and context: This paper could benefit from more discussion of diseases in which heme is a driver of clinical complications, and a more complete discussion of therapeutics already in this space.

--- Originality and significance. Our group (Li Lab) and our collaborators' group (Shaul & Mineo Lab) have studied SR-BI biology for over 25 years. To our best knowledge, no published work has systematically evaluated SR-BI's function as a *direct* free heme scavenger. We respectfully invite the reviewer to provide references supporting the assertion that this function of SR-BI has been known "for over a decade," as such citations would help contextualize our findings. Our work identifies and mechanistically validates this previously unrecognized function of SR-BI, which we believe represents a substantial advance.

--- Data & methodology. The acute hemin challenge model was intentionally used to isolate SR-BI–dependent mechanisms under controlled conditions. Chronic hemolytic models alone cannot resolve receptor-specific contributions due to compensatory pathways and variable heme flux. Our adenoviral SR-BI overexpression experiments in SCD-Berk mice demonstrate that augmenting SR-BI is sufficient to confer protection even in the context of ongoing hemolysis, providing proof-of-principle translational relevance. The observation that SR-BI is downregulated in SCD is indeed intriguing; however, detailed exploration of disease-specific regulatory mechanisms is beyond the scope of this mechanistic study and represents an important future direction.

--- Suggested improvements. We agree that pharmacologic SR-BI induction using agents with established human safety profiles would be a valuable next step. However, such studies require dedicated pharmacokinetic, toxicity, and dosing optimization that extend beyond the scope of this discovery-driven report. Our current work establishes the mechanistic foundation necessary to justify such translational efforts.

Regarding RNA-seq, our transcriptomic analysis was intentionally performed in SR-BI–deficient mice to define pathways that become dysregulated specifically due to loss of SR-BI, without the confounding influence of disease-specific transcriptional programs.

--- Clarity and context. We appreciate reviewer's suggestion. We recently published a review article, which provides a comprehensive overview of current therapeutic approaches for treating SCD intravascular hemolysis. Following suggestion, we now reference this paper in our discussion[28].

References

1. Benavides BS, Valandro S, Cioloboc D, Taylor AB, Schanze KS, Kurtz DM, Jr. Structure of a Zinc Porphyrin-Substituted Bacterioferritin and Photophysical Properties of Iron Reduction. *Biochemistry*. 2020;59:1618-1629. doi: 10.1021/acs.biochem.9b01103
2. Chen-Roetling J, Cai Y, Lu X, Regan RF. Hemin uptake and release by neurons and glia. *Free Radic Res*. 2014;48:200-205. doi: 10.3109/10715762.2013.859386
3. Worthington MT, Cohn SM, Miller SK, Luo RQ, Berg CL. Characterization of a human plasma membrane heme transporter in intestinal and hepatocyte cell lines. *Am J Physiol Gastrointest Liver Physiol*. 2001;280:G1172-1177. doi: 10.1152/ajpgi.2001.280.6.G1172
4. Huynh C, Yuan X, Miguel DC, Renberg RL, Protchenko O, Philpott CC, Hamza I, Andrews NW. Heme uptake by Leishmania amazonensis is mediated by the transmembrane protein LHR1. *PLoS Pathog*. 2012;8:e1002795. doi: 10.1371/journal.ppat.1002795
5. Shayeghi M, Latunde-Dada GO, Oakhill JS, Laftah AH, Takeuchi K, Halliday N, Khan Y, Warley A, McCann FE, Hider RC, et al. Identification of an intestinal heme transporter. *Cell*. 2005;122:789-801. doi: 10.1016/j.cell.2005.06.025
6. Hvidberg V, Maniecki MB, Jacobsen C, Hojrup P, Moller HJ, Moestrup SK. Identification of the receptor scavenging hemopexin-heme complexes. *Blood*. 2005;106:2572-2579. doi: 10.1182/blood-2005-03-1185
7. Dutra FF, Alves LS, Rodrigues D, Fernandez PL, de Oliveira RB, Golenbock DT, Zamboni DS, Bozza MT. Hemolysis-induced lethality involves inflammasome activation by heme. *Proc Natl Acad Sci U S A*. 2014;111:E4110-4118. doi: 10.1073/pnas.1405023111
8. Pfefferle M, Ingoglia G, Schaer CA, Yalamanoglu A, Buzzi R, Dubach IL, Tan G, Lopez-Cano EY, Schulthess N, Hansen K, et al. Hemolysis transforms liver macrophages into antiinflammatory erythrophagocytes. *J Clin Invest*. 2020;130:5576-5590. doi: 10.1172/JCI137282
9. Marcero JR, Piel Iii RB, Burch JS, Dailey HA. Rapid and sensitive quantitation of heme in hemoglobinized cells. *Biotechniques*. 2016;61:83-91. doi: 10.2144/000114444
10. Dunaway LS, Loeb SA, Petrillo S, Tolosano E, Isakson BE. Heme metabolism in nonerythroid cells. *The Journal of biological chemistry*. 2024;300:107132. doi: 10.1016/j.jbc.2024.107132
11. Uchida N, Haro-Mora JJ, Demirci S, Fujita A, Raines L, Hsieh MM, Tisdale JF. High-level embryonic globin production with efficient erythroid differentiation from a K562 erythroleukemia cell line. *Exp Hematol*. 2018;62:7-16 e11. doi: 10.1016/j.exphem.2018.02.007

12. Morse D, Lin L, Choi AM, Ryter SW. Heme oxygenase-1, a critical arbitrator of cell death pathways in lung injury and disease. *Free Radic Biol Med*. 2009;47:1-12. doi: 10.1016/j.freeradbiomed.2009.04.007
13. Reiter TA, Pang B, Dedon P, Demple B. Resistance to nitric oxide-induced necrosis in heme oxygenase-1 overexpressing pulmonary epithelial cells associated with decreased lipid peroxidation. *The Journal of biological chemistry*. 2006;281:36603-36612. doi: 10.1074/jbc.M602634200
14. Grunenwald A, Peliconi J, Lavergne J, Revel M, Voilin E, Robe-Rybkine T, Crambert G, Dimitrov JD, Blanc-Brude O, Roumenina LT. Transcriptomic atlas reveals organ-specific disease tolerance in sickle cell mice. *Blood Adv*. 2025;9:1405-1419. doi: 10.1182/bloodadvances.2024013435
15. Sparkenbaugh EM, Chantrathammachart P, Wang S, Jonas W, Kirchhofer D, Gailani D, Gruber A, Kasthuri R, Key NS, Mackman N, et al. Excess of heme induces tissue factor-dependent activation of coagulation in mice. *Haematologica*. 2015;100:308-314. doi: 10.3324/haematol.2014.114728
16. Chadwick AC, Jensen DR, Hanson PJ, Lange PT, Proudfoot SC, Peterson FC, Volkman BF, Sahoo D. NMR Structure of the C-Terminal Transmembrane Domain of the HDL Receptor, SR-BI, and a Functionally Relevant Leucine Zipper Motif. *Structure*. 2017;25:446-457. doi: 10.1016/j.str.2017.01.001
17. Powers HR, Sahoo D. SR-B1's Next Top Model: Structural Perspectives on the Functions of the HDL Receptor. *Curr Atheroscler Rep*. 2022;24:277-288. doi: 10.1007/s11883-022-01001-1
18. Camponeschi F, Gallo A, Piccioli M, Banci L. The long-standing relationship between paramagnetic NMR and iron-sulfur proteins: the mitoNEET example. An old method for new stories or the other way around? *Magn Reson (Gott)*. 2021;2:203-221. doi: 10.5194/mr-2-203-2021
19. Neculai D, Schwake M, Ravichandran M, Zunke F, Collins RF, Peters J, Neculai M, Plumb J, Loppnau P, Pizarro JC, et al. Structure of LIMP-2 provides functional insights with implications for SR-BI and CD36. *Nature*. 2013;504:172-176. doi: 10.1038/nature12684
20. Hu J, Zhang ZH, Shen WJ, Nomoto A, Azhar S. Differential Roles of Cysteine Residues in the Cellular Trafficking, Dimerization, and Function of the High-Density Lipoprotein Receptor, SR-BI. *Biochemistry*. 2011;50:10860-10875. doi: 10.1021/bi201264y
21. Guo L, Chen M, Song ZQ, Daugherty A, Li XA. C323 of SR-BI is required for SR-BI-mediated HDL binding and cholesteryl ester uptake. *Journal of Lipid Research*. 2011;52:2272-2278. doi: 10.1194/jlr.M019091
22. Yu M, Lau TY, Carr SA, Krieger M. Contributions of a disulfide bond and a reduced cysteine side chain to the intrinsic activity of the high-density lipoprotein receptor SR-BI. *Biochemistry*. 2012;51:10044-10055. doi: 10.1021/bi301203x
23. Rasmussen JT, Berglund L, Rasmussen MS, Petersen TE. Assignment of disulfide bridges in bovine CD36. *Eur J Biochem*. 1998;257:488-494. doi: 10.1046/j.1432-1327.1998.2570488.x
24. Hu J, Zhang Z, Shen WJ, Nomoto A, Azhar S. Differential roles of cysteine residues in the cellular trafficking, dimerization, and function of the high-density lipoprotein receptor, SR-BI. *Biochemistry*. 2011;50:10860-10875. doi: 10.1021/bi201264y

25. Cain DM, Vang D, Simone DA, Hebbel RP, Gupta K. Mouse models for studying pain in sickle disease: effects of strain, age, and acuteness. *Br J Haematol*. 2012;156:535-544. doi: 10.1111/j.1365-2141.2011.08977.x
26. Vincent L, Vang D, Nguyen J, Gupta M, Luk K, Ericson ME, Simone DA, Gupta K. Mast cell activation contributes to sickle cell pathobiology and pain in mice. *Blood*. 2013;122:1853-1862. doi: 10.1182/blood-2013-04-498105
27. Federti E, Matte A, Recchiuti A, Garello F, Ghigo A, El Nemer W, Terreno E, Amoresano A, Mattoscio D, Turrini F, et al. In Humanized Sickle Cell Mice, Imatinib Protects Against Sickle Cell-Related Injury. *Hemasphere*. 2023;7:e848. doi: 10.1097/HS9.0000000000000848
28. Xue J, Li XA. Therapeutics for sickle cell disease intravascular hemolysis. *Front Physiol*. 2024;15:1474569. doi: 10.3389/fphys.2024.1474569
